# MSK-Mediated Phosphorylation of Histone H3 Ser28 Couples MAPK Signalling with Early Gene Induction and Cardiac Hypertrophy

**DOI:** 10.3390/cells11040604

**Published:** 2022-02-09

**Authors:** Emma L. Robinson, Faye M. Drawnel, Saher Mehdi, Caroline R. Archer, Wei Liu, Hanneke Okkenhaug, Kanar Alkass, Jan Magnus Aronsen, Chandan K. Nagaraju, Ivar Sjaastad, Karin R. Sipido, Olaf Bergmann, J. Simon C. Arthur, Xin Wang, H. Llewelyn Roderick

**Affiliations:** 1 Laboratory of Experimental Cardiology, Department of Cardiovascular Sciences, KU Leuven, B-3000 Leuven, Belgium; saher@wellowise.com (S.M.); chandan.kadurnagaraju@kuleuven.be (C.K.N.); karin.sipido@kuleuven.be (K.R.S.); 2 Department of Cardiology, Cardiovascular Research Institute Maastricht, Maastricht University, 6229 ER Maastricht, The Netherlands; 3 Epigenetics and Signalling Programmes, Babraham Institute, Cambridge CB22 3AT, UK; faye.drawnel@roche.com (F.M.D.); caroline.archer@astrazeneca.com (C.R.A.); hanneke.okkenhaug@babraham.ac.uk (H.O.); 4 Roche Pharma Research and Early Development, Roche Innovation Center Basel, F. Hoffmann-La Roche Ltd., 4070 Basel, Switzerland; 5 Faculty of Biology, Medicine and Health, University of Manchester, Manchester M13 9PT, UK; Wei.Liu@manchester.ac.uk (W.L.); xin.wang@manchester.ac.uk (X.W.); 6 Department of Oncology and Pathology, Karolinska Institute, SE-17177 Stockholm, Sweden; kanar.alkass@ki.se; 7 Institute for Experimental Medical Research, Oslo University Hospital, University of Oslo, 0450 Oslo, Norway; j.m.aronsen@medisin.uio.no (J.M.A.); ivar.sjaastad@medisin.uio.no (I.S.); 8 Bjørknes College, Oslo University, 0456 Oslo, Norway; 9 KG Jebsen Center for Cardiac Research, University of Oslo, 0450 Oslo, Norway; 10 Cell and Molecular Biology, Biomedicum, Karolinska Institutet, SE-17177 Stockholm, Sweden; olaf_bergmann@tu-dresden.de; 11 Division of Immunology and Cell Signalling, School of Life Sciences, University of Dundee, Dundee DD1 5EH, UK; j.s.c.arthur@dundee.ac.uk

**Keywords:** cardiomyocyte, MSK, phosphorylated histone 3 serine 28, immediate early genes, hypertrophy

## Abstract

Heart failure is a leading cause of death that develops subsequent to deleterious hypertrophic cardiac remodelling. MAPK pathways play a key role in coordinating the induction of gene expression during hypertrophy. Induction of the immediate early gene (IEG) response including activator protein 1 (AP-1) complex factors is a necessary and early event in this process. How MAPK and IEG expression are coupled during cardiac hypertrophy is not resolved. Here, in vitro, in rodent models and in human samples, we demonstrate that MAPK-stimulated IEG induction depends on the mitogen and stress-activated protein kinase (MSK) and its phosphorylation of histone H3 at serine 28 (pH3S28). pH3S28 in IEG promoters in turn recruits Brg1, a BAF60 ATP-dependent chromatin remodelling complex component, initiating gene expression. Without MSK activity and IEG induction, the hypertrophic response is suppressed. These studies provide new mechanistic insights into the role of MAPK pathways in signalling to the epigenome and regulation of gene expression during cardiac hypertrophy.

## 1. Introduction

Cardiovascular diseases (CVDs) are the leading cause of mortality and morbidity worldwide [1]. While cardiac hypertrophy is initially an adaptive response to increased workload or stress, when induced by pathological cues, the response can decompensate, resulting in a decline in cardiac function and progression to heart failure. As cardiomyocytes (CM) are terminally differentiated, hypertrophy of the cardiac muscle is mediated by growth of CM and not through their proliferation [2].

Signalling pathways downstream of G-protein-coupled receptors (GPCR), such as endothelin-1 (ET-1), angiotensin II (AngII) and adrenergic receptors, play a fundamental role in the induction of pathological hypertrophic remodelling [3,4,5,6]. Mitogen-activated protein kinases (MAPK) are of particular importance in mediating the pro-hypertrophic actions, GPCR signalling contributing to regulation of protein synthesis, cell survival, metabolism and gene transcription [6,7]. MAPKs fall into four major families—the extracellular regulated kinases 1 and 2 (ERK1/2), p38 MAPK, c-Jun N-terminal kinases 1 and two (JNK1/2) and ERK5 [6,7]. All limbs of this kinase family are involved in regulating hypertrophic remodelling [6,8,9]. During cardiac hypertrophy, MAPK pathways regulate transcription via phosphorylation-dependent modulation of transcription factors such as NFAT, Elk, SRF and GATA4 [10]. Preceding the induction of expression of genes associated with the hypertrophic state, hypertrophic cues such as pressure overload and exposure to neurohormonal agonists stimulate a MAPK-dependent rapid activation of an immediate early gene (IEG) response [11,12,13,14]. This IEG response is initiated through phosphorylation-dependent activation of the activator protein 1 (AP-1) transcription factor [14,15,16,17]. The AP-1 transcription factor is a dimeric complex formed by members of the FOS (c-FOS, FOSB, FRA-1 and FRA-2), Jun (JUNB, JUND and c-JUN), activating transcription factor (ATF; ATFa, ATF2, LRF1/ATF3, ATF4 and B-ATF) and MAF families of basic-leucine zipper transcription factors, which are themselves induced as part of the IEG response [17,18,19]. AP-1 transcription factors also act though forming heteromeric interactions with other transcription factors such as NFκB and NFAT, with known roles in CM [20,21]. In addition to showing hypertrophy-related alterations in their activation and expression, a functional role for AP-1 factors in cardiac hypertrophic remodelling is described. While AP-1 factors are generally required for hypertrophic responses in vitro [22,23], in vivo roles of different AP-1 factors are more complex. For example, in vivo deletion of *JunD* or of *c-Jun* results in a loss of the initial adaptive response to hypertrophic stimuli and an exacerbation of the deleterious remodelling to pressure overload [24,25,26]. Contributing to this phenotype is a reduced upregulation of sarcomeric proteins, enhanced CM apoptosis and fibrosis. *c-Fos* deletion is without effect, however [26]. Contrastingly, JunD overexpression results in ventricular dilation and reduced contractility [25], although in vitro studies suggest that JunD suppresses hypertrophic responses by inhibiting the action of c-Fos and c-Jun [22]. Taken together, these reports demonstrate the important and complex functions of AP-1 transcription factors in hypertrophic remodelling, particularly in the early adaptive responses to pathological cues.

Although ERK activation is highly correlated with induction of IEG expression during CM hypertrophic responses [11,27], the mechanism linking these events is not resolved. In other tissues however, IEG expression is induced following MAPK pathway activation via a mechanism involving phosphorylation of serines 10 and 28 (H3S10 and H3S28) in the histone H3 NH_2_-terminal tail at IEG loci, termed the nucleosomal response [28,29]. Phosphorylated histone H3 creates a permissive environment for induction of transcription through recruitment of scaffolding proteins such as 14-3-3 family members, transcriptional regulators and chromatin remodelling factors [30]. The lack of consensus sites in histone H3 for phosphorylation by ERK1/2 indicates that it is not the responsible kinase. Rather, during the nucleosomal response, histone H3 is phosphorylated by the mitogen and stress-activated kinases (MSK1/2) [30,31,32,33]. MSKs are nuclear-localised kinases that are activated by an initial phosphorylation by upstream MAPK including ERK1/2 and subsequent autophosphorylation [34,35]. MSK1 and the highly homologous kinase MSK2 are both expressed in the heart and are activated in response to hypertrophic stimuli [36,37,38]. While the functional role of MSK1/2 in CM hypertrophic responses has been tested in vitro, the substantial off-target effects of the MSK inhibitors used on other kinases such as RSK2, PKCs and S6 kinase, which themselves are involved in hypertrophic signalling, preclude its establishment as a contributor to the CM hypertrophic response [39,40,41]. Further, whether MSK participates in cardiac hypertrophy in vivo and in human is not determined. Establishing the contribution and underlying mechanisms of MSK in CM hypertrophy remains the key, however, to understanding how CM responds to hypertrophic stimuli and the mechanism by which MAPK pathways and AP-1 factors elicit their effects in this process.

Here, using a combination of molecular and pharmacological approaches, including *Msk1/2* knockout mice, we determined that MSK1/2 activated downstream of ERK1/2 was required for CM hypertrophic responses both in vitro and in vivo. MSKs act through promoting the phosphorylation of histone H3S28 (pH3S28), which in turn recruits AP-1 factors, together with the chromatin remodeller BRG-1 to IEG promoters, inducing transcription of IEG and CM-hypertrophy-associated genes. Activation of this pathway also contributed to CM hypertrophy induction. Notably, the activation and role of the ERK1/2-MSK1/2-pH3S28 molecular axis was conserved in human samples, thereby supporting its relevance to human disease.

Together, our data identify MSK as key and missing components in the signalling pathway that transduces the activation of GPCRs by pathological pro-hypertrophic mediators in CM to the induction of hypertrophic remodelling.

## 2. Materials and Methods

### 2.1. Reagents

Chemicals were from Merck (Darmstadt, Germany) and molecular biology reagents were from (Thermo Fisher Scientific, Bedford, MA, USA), unless stated otherwise. Tables of antibodies and primers used in this study are included in Supplementary Tables S1 and S2, respectively.

### 2.2. Animal Experiments

Experiments involving animals were conducted in accordance with the guidelines from the Home Office code of practice for humane killing of animals under Schedule 1 of the Animals (Scientific Procedures) Act 1986 (UK) and in accordance with the European Directive 2010/63/EU and approved by the Ethical Committee for Animal Experiments of the KU Leuven (Belgium) under license number P055/2017 (approval date 16 March 2017) and P024/2015 (approval date 01 January 2015). The *Msk1/2* null animals have been previously described [42,43]. Hypertrophic remodelling was induced in 8–10-week-old male mice animals by administration of isoproterenol (Iso, Merck) at 10 mg/kg/day for one week via osmotic mini-pumps (Alzet) implantation as previously described and under project license P3A97F3D1 [5]. Jugular vein infusion of ET-1 and Iso was performed as previously described [11] and explained in greater detail later. 

### 2.3. Echocardiography

Mice were anaesthetised with Avertin (200 mg/kg). Cardiac function was assessed by transthoracic 2D M-mode echocardiography using an Acuson Sequoia C256 ultrasound system (Siemens, Munich, Germany) as previously described [5].

### 2.4. Preparation of Neonatal Rat Ventricular Cardiomyocytes (NRVMs)

Primary neonatal rat ventricular CM (NRVMs) were isolated from 3–4-day-old male and female Wistar pups and cultured as described previously [44]. Cultures were >95% pure. Adenoviral infections were as previously described [44]. Agonist treatments diluted in serum-free medium were applied 24 h post-infection with adenovirus. Endothelin-1 (ET-1), Iso and PD184352 (PD) were used at final concentrations of 100 nM, 10 nM and 1 µM, respectively. PD was applied for 30 min prior to hypertrophic agonist application (ET-1/Iso). Control cellular experiments (no treatment) were treated with the same volume of vehicle only (DMSO for ET-1 and PD).

### 2.5. Isolation and Culture of Adult Rat Ventricular Cardiomyocytes (ARVMs)

Male Wistar rats (Harlan; ~200 g) were anaesthetised by CO_2_ inhalation and sacrificed by cervical dislocation. ARVMs isolation by Langendorff and collagenase digestion, culture and adenoviral infections were as previously described [45]. For experiments involving acute stimulation with ET-1 and Iso, cells in HEPES-buffered Tyrode’s solution (in mmol/L: NaCl 137, KCl 5.4, MgCl_2_ 0.5, CaCl_2_ 1.8, NaHEPES 11.8 and glucose 10; pH 7.4) were plated onto laminin-coated 8-well Nunc cover glasses cells (25 µg/mL), allowed to attach for 1 h at 37 °C, after which the Tyrode’s solution was replaced with Tyrode’s solution containing DMSO vehicle or 10 nM PD. After 20 min, buffer was exchanged for Tyrode’s solution containing 100 nM ET-1 or 10 nM Iso ± PD. After 15 min, dishes were placed on ice and processed for immunostaining and imaging.

### 2.6. Isolation of Human Ventricular Cardiomyocytes

Cardiomyocytes were isolated as previously described from non-failing heart tissue samples obtained from donor hearts not suitable for transplantation [46]. Donor human tissue was collected under a study protocol approved by the ethical committee of UZ Leuven (S58824), which conformed to the Helsinki declaration and was conducted in accordance with the prevailing national and European Union regulations on the use of human tissues. Donor information is displayed in Supplementary Table S3. After isolation, the cells were allowed to recover for 1 h before starting experiments or fixation. For experiments involving stimulation with Iso or ET-1, CM was plated onto poly-L-lysine-coated 8-well Nunc cover glasses and allowed to attach for 1 h at 37 °C. After this period, the Tyrode’s solution was replaced with Tyrode’s solution containing either DMSO vehicle or 1 µM PD. After 20 min, buffer was exchanged for Tyrode’s solution containing 100 nM ET-1 or 10 nM Iso ± PD. After 15 min, dishes were placed on ice and processed for immunostaining and imaging.

### 2.7. Isolation of Human Cardiomyocyte Nuclei

Nuclei from post-mortem left ventricular tissue were isolated and flow sorted according to pericentriolar material 1 (PCM-1) staining as previously described [47]. In total, 500,000 nuclei were sorted into 1 mL TRIzol reagent for RNase inhibition prior to RNA isolation. Human LV samples were obtained from the KI Donatum, Karolinska Institutet, Stockholm, Sweden, with permission for the analysis of human tissue for research purposes granted by the Regional Ethics Committee in Stockholm, Sweden. Donor information from which LV CM nuclei were isolated is displayed in Supplementary Table S4.

### 2.8. High Content Analysis of NRVM Hypertrophy

Analysis of surface area of NRVM was carried out as previously described with modifications [44,47]. Briefly, NRVMs for immunofluorescence were cultured and fixed in black 96-well imaging microplates (BD Biosciences, Franklin Lakes, NJ, USA). NRVMs were immunostained with primary antibodies against α-Actinin (α-Act) and atrial natriuretic factor (ANF) and detected using Alexa Fluor 488 and 568-coupled secondary antibodies (Supplementary Table S1). After immunostaining, nuclei were labelled with Hoescht (1 µg/mL in PBS for 20 min). Images were captured using a BD Pathway 855 high-content imaging system and Attovision software. Cell planimetry was performed using ImageJ by drawing around the edge of the cells (NIH). At least 400 cells from three independent experiments were analysed. These images were also used for quantification of ANF protein expression as determined by counting the number of NRVM exhibiting a peri-nuclear ring of ANF.

### 2.9. Confocal Imaging of Immunostained NRVM and ARVM

NRVMs were cultured in 16-well chamber slides (Nunc) and immunostained as for high-content imaging. Slides were mounted onto coverslips using VECTASHIELD Mounting Medium containing DAPI and sealed with clear nail varnish.

For immunostaining of ARVMs and isolated human CMs, cells were fixed and permeabilised in ice-cold 100% methanol and incubated at −20 °C for 10 min. Methanol was washed from the coverslips twice with PBS and further permeabilisation was performed by the addition of 0.5 mL ice-cold 100% acetone and incubation at −20 °C for 1 min. Following an additional two washes in PBS, antibody labelling was performed as described for NRVM. To further reduce background staining, blocking and secondary antibody buffers contained 1% BSA in addition to goat serum. Images were captured using a Nikon A1R confocal microscope equipped with a Plan Fluor DIC H N 40x/1.3 NA oil immersion objective (human CM and ARVM treated with hypertrophic agonists) or using an Olympus FV1000 point scanning microscope attached to an Olympus IX81, equipped with a 40x/1.3 NA UPlanFI oil immersion objective (ARVM expressing MSK constructs).

### 2.10. Confocal Imaging of Immunostained Cardiac Tissue Sections

Immunofluorescence analysis of cardiac sections was performed as previously described [47]. Sections were thawed and rehydrated in phosphate-buffered saline (PBS) for 5 min, followed by 15 min fixation in 4% paraformaldehyde (PFA). After three washes (5 min each) in PBS, sections were permeabilised for 30 min in 0.2% Triton X-100 in PBS (PBS-TX), then washed twice with PBS. Non-specific protein-binding sites were blocked by incubation in PBS-TX containing 3% BSA, 5% goat serum or 5% Chemibloc for 1 h. Sections were subsequently incubated overnight at 4 °C in blocking buffer with primary antibodies as per Supplementary Table S1. Samples were washed extensively in PBS-TX, and incubated with Alexa Fluor-conjugated secondary antibodies at 1:500 in PBS-TX for 1 h at room temperature. For detection of apoptosis in tissue sections, the TACS^®^ 2 TdT-DAB In Situ Apoptosis Detection Kit was used (Bio-Techne Ltd, Abingdon, UK). Prior to imaging, sections were mounted in VECTASHIELD with DAPI (Vector Labs, Burlingame, CA, USA). Images were captured using a Nikon A1R confocal microscope equipped with a Plan Fluor DIC H N 40x/1.3 NA oil immersion objective. Image stacks were collected over a 2 µm stack thickness (0.2 µm z-step). Image stacks were analysed with Volocity Image analysis software (version 6.2.1, Perkin Elmer, Hopkinton, MA, USA). Intensity of labelling of targets of interest was quantitated specifically in cardiac myocyte nuclei, which were identified as staining positive for DAPI and for either PCM-1 or Nesprin, as previously described [41].

### 2.11. Picro Sirius Red Staining for Fibrosis Analysis

First, 10 µm thick sections were cut from OCT embedded tissue as above. Subsequently, sections were rehydrated and stained for collagen using a Picro Sirius red staining kit (PolySciences, Hirschberg an der Bergstraße, Germany). After staining, sections were mounted in dibutylphthalate polystyrene xylene mounting medium. Images were acquired using a Zeiss Axioplan microscope configured with an Axiocam HrC camera. Polarization microscopy was performed on the Sirius-red-stained sections to visualize collagen type I and III based on the birefringence properties of collagen. The degree of fibrosis was quantified using Zeiss Axiovision analysis software (Version 4.6).

### 2.12. Histone Isolation by Acid Extraction

NRVMs in 6-well dishes were washed once in ice-cold PBS. Then, 0.5 mL of fresh ice-cold PBS was added to each well, the cells scraped and placed in pre-chilled 1.5 mL tubes. Cells were pelleted by centrifugation (10 min, 300× *g*, 4 °C). PBS was removed and the pellet resuspended in 1 mL hypotonic lysis buffer. The resuspended cells were incubated on a rotator at 4 °C, 30 rpm for 30 min. At the end of this incubation, intact nuclei were pelleted by centrifugation (10 min, 10,000× *g*, 4 °C). Nuclei were then resuspended in 400 µL 0.2 M H_2_SO_4_ by pipetting and vortexing. Histones were acid extracted overnight at 4 °C on a rotator at 30 rpm. Following acid extraction, nuclear debris was removed by centrifugation (10 min, 16,000× *g*, 4 °C) and the supernatant containing isolated histones was transferred to a pre-chilled tube. To precipitate proteins, 100% trichloroacetic acid was added to the supernatant in a drop-wise manner to achieve a final concentration of 25%. The tube was gently inverted and then incubated on ice for 6 h. At the end of this period, precipitated proteins were recovered by centrifugation (10 min, 16,000× *g*, 4 °C). The supernatant was aspirated and acid removed from the tube by washing the pellet in 300 µL ice-cold acetone. After centrifugation (5 min, 16,000× *g*, 4 °C) and removal of the supernatant, the acetone wash and spin were repeated. Finally, the supernatant was gently removed and the pellet air-dried for 20 min at room temperature. The dried pellet was resuspended in 50 µL water and incubated overnight at 4 °C on a rotator at 30 rpm to maximise protein solubilisation.

### 2.13. Immunoblot Analysis

Immunoblotting was performed as previously reported with minor modifications [47]. First, 10–30 µg of protein lysates was prepared under reducing conditions with LDS sample buffer (Thermo Fisher Scientific) containing 2.5% β-mercaptoethanol and boiled at 95 °C for 5 min. Proteins were resolved on pre-cast 4–12% NuPAGE 1.5 mm 10-well SDS gels using MOPS running buffer (Thermo Fisher Scientific). Novex pre-stained sharp protein markers were used (Thermo Fisher Scientific).

For detection of ERK or MSK, proteins were transferred to a PVDF membrane (0.45 µm; Merck). For detection of histone H3 and its modified forms, proteins were transferred to nitrocellulose (0.2 µm, Whatman/Cytiva, Marlborough, MA, USA). Proteins were detected with appropriate primary antibodies and HRP-conjugated secondary antibodies (Supplementary Table S1). Immunoreactive bands were detected by enhanced chemiluminescence (Thermo Fisher Scientific).

### 2.14. Reverse Transcription Quantitative PCR (RT-qPCR)

RNA was isolated from NRVMs and ARVMs using the RNeasy Micro Kit (Qiagen) and DNA was removed by an on-column DNA digestion step. RNA was isolated from adult rat LV tissue, *Msk1/2* knockout (KO) mouse LV tissue and human CM using TRIzol reagent. Then, 500–750 ng RNA was reverse transcribed using Superscript II. Primer sequences were as previously described, unless otherwise indicated (Supplementary Table S2), and were designed to span intron-exon boundaries to avoid amplification of genomic DNA [44]. Stable reference genes for each experiment were selected from a wider panel using the GeNorm method [48]. Three or four reference genes were used for normalisation of gene expression. Final primer concentration was 200 nM for all targets. Reactions were performed on a LightCycler^®^ 480 System (Roche, Basel, Switzerland) or on a CFX384 (Bio-Rad, Hercules, CA, USA) in a 384-well format using Platinum SYBR Green qPCR SuperMix-UDG. A mean of triplicate reactions was used per sample. Expression analysis was carried out using the comparative ∆Ct method as described [49].

### 2.15. Chromatin-Immunoprecipitation (ChIP)

ChIP was performed according to standard conditions. To NRVMs in 6-well dishes in culture medium, formaldehyde was added to a concentration of 1% and incubated for 10 min on a rocking platform at room temperature. Cross-linking was terminated by the addition of 125 mM glycine for 10 min at room temperature. Nuclei were isolated by hypotonic lysis of cells after which chromatin was extracted. Cross-linked chromatin was fragmented by sonication using a pre-chilled Diagenode Bioruptor on the high-power setting for three × 5 min cycles of 30 s ‘on’, 30 s ‘off’. The sonication protocol produced fragments predominantly below 500 bp. After removal of debris by centrifugation (10 min, 12,000× *g*, 4 °C), the supernatant (sonicated chromatin) was processed for immunoprecipitation. Proteins of interest were precipitated using antibodies pre-conjugated to Dynabeads Protein A (Thermo Fisher Scientific). Antibodies used for ChIP are indicated in Supplementary Table S1. To this end, beads were washed in 4 changes of wash buffer and collected using a DynaMag Magnet. Per ChIP, 10 µL washed beads and 5 µg of antibody against BRG1or of phosphorylated histone H3S28 were added to 90 µL wash buffer and incubated for 2 h at 40 rpm on a rotator at 4 °C. For the negative control ChIP, beads were used in the absence of specific antibody.

Prior to IP, wash buffer was removed from pre-prepared antibody-bead complexes and 200 µL chromatin was added to each tube. Then, 200 µL chromatin was reserved from each experimental condition as an input sample. Chromatin was incubated with the antibody-bead complexes overnight at 4 °C on a 40 rpm rotator after which unbound chromatin was washed from the beads. Beads were washed ×2 in wash buffer, followed by one wash in high-salt wash buffer (wash buffer + with 500 mM NaCl) and finally ×2 washes in TE buffer. The solution was transferred to a fresh tube and TE buffer was removed from the beads. Elution of chromatin from the beads and protein digest with proteinase K were combined into one step. Chromatin was then eluted from beads and digested with proteinase K. Input samples were processed in parallel with the ChIP samples. DNA was purified from each supernatant using the QIAEX II Gel extraction kit (Qiagen) following the manufacturer’s protocol. DNA was eluted in 40 µL buffer EB and stored at −20 °C.

Precipitated DNA for each experimental condition and antibody was quantified by qPCR using SYBR-GreenER in 12.5 µL reactions performed in triplicate (Thermo Fisher Scientific). Sequences of primers used are given in Supplementary Table S2. Primer-binding sites were selected that encompassed predicted transcription factor binding sites. qPCR was performed using a CFX96 (Bio-Rad) or Roche Light Cycler480 real-time PCR instrument and cycling parameters were taken from the manufacturer’s instructions for SYBR-GreenER (Thermo Fisher Scientific). Ct values from triplicate technical replicates from each sample were averaged to generate SampleCT and InputCt values. The values were analysed by expressing enrichment of the immunoprecipitated DNA for each antibody as a percentage of the input sample for the relevant experimental condition. ChIPs were repeated on at least three independent experimental samples.

### 2.16. Jugular Vein Infusion of Endothelin-1/Isoproterenol in Wistar Rat

Experimental protocols were approved by the local ethical committee (Ethische Commissie, Dierproeven, KU Leuven), under license number P055/2017 and were performed as previously described [11]. In total, 250–300 g Wistar (RccHan:WIST) male rats were obtained from Harlan (NL).

The cannula was attached to a 5 mL syringe and a dispensing pump (Harvard Apparatus) dispensing the required volume (300–500 µL) over a 15 min period. A slow steady release of the dosage in this manner was required to reduce the acute vasoconstrictive effect of a single rapid injection of the same dosage. ET-1 (Millipore) was administered at a final dosage of 1000 ng/kg and Iso (Merck) at 50 µg/kg. Final working concentrations prepared in sterile saline and vehicle-only controls (Ctrl) were administered at the same volume of sterile saline over a 15 min period. Rats were sacrificed by cervical dislocation and the heart was immediately removed for dissection. Whole hearts were removed and placed in ice-cold PBS briefly to remove excess blood, dissected using a sterile surgical scalpel in PBS on ice and weighed on a microbalance before snap-freezing in liquid nitrogen and being stored at −80 °C.

### 2.17. Adenoviral Methods

Adenoviruses were produced and amplified in HEK293 cells and purified as previously described [44]. Adenoviruses to express the WT and catalytically dead D565A mutant (DN) of MSK1 were generated using the AdEasy method by sub-cloning the cDNA for MSK1 or its mutant from a pCMV5 backbone (kindly provided by Prof D Alessi, University of Dundee) into pShuttle CMV [39]. PacI-digested recombinant plasmids were transfected into HEK293 cells and crude adenovirus was harvested after 10–14 days. Adenoviruses for dominant negative (DN)-Jun and AP-1 luciferase were purchased from Vector Biolabs (Malvern, PA, USA). All viruses were amplified in HEK293 cells, purified using the Vivapure Adenopack 100 (Sartorius, Gottingen, Germany) and titrated by end-point dilution in HEK293 cells.

### 2.18. Analysis of Luciferase Reporter Activity

The AP-1 luciferase reporter was expressed using an adenoviral vector and luciferase activity was determined using a luciferase assay kit from Promega (Madison, WI, USA) as previously described [42].

### 2.19. Small Interfering RNA (siRNA) Knockdown

Stealth™ siRNAs were purchased from Invitrogen. To achieve sufficient knockdown of *Msk1* or *Brg1*, two siRNAs targeting different regions of the target mRNA were selected. Medium GC-content non-silencing siRNA was transfected as a negative control. Transfections were performed at the onset of the serum starvation period using Dharmafect I and Accell medium (Dharmacon, Horizon Discovery, Cambridge, UK). To transfect NRVM cultured in 12-well dishes, 200 pmol siRNA duplexes were made up to 100 µL total volume in Accell media and the solution was mixed by pipetting. In a separate tube, 6 µL Dharmafect I was added to 94 µL Accell medium and mixed. After incubation for 5 min at room temperature, the tubes were combined, mixed and incubated for a further 20 min at room temperature. During this incubation, NRVM culture medium was replaced with 800 µL pre-warmed Accell medium. At the end of the 20 min incubation, transfection complexes were added to the cultures in a drop-wise manner and incubated with the cells for 6 h. Post-transfection, Accell medium was replaced with fresh maintenance medium and the remainder of the serum starvation period was carried out.

### 2.20. Statistical Analysis

Data were collated in Microsoft Excel and statistical analysis was performed using GraphPad Prism v7.0 or v8.0. Data are presented as the mean of at least three independent experiments ± the standard error of the mean (SEM). The number of independent experiments for each figure is indicated in the figure legend. For comparison between two groups, *p*-values were calculated using the unpaired two-tailed *t*-test. To calculate *p*-values for data comparing three or more groups, one-way ANOVA with Bonferroni’s multiple comparison test for *p* value correction was used. *p*-values less than 0.05 were accepted as significant. Individual (adjusted) *p* values are indicated on the figures.

## 3. Results

### 3.1. Endothelin-1 Stimulates ERK-Dependent Phosphorylation of Histone H3 Serine 28

**Figure 1:**
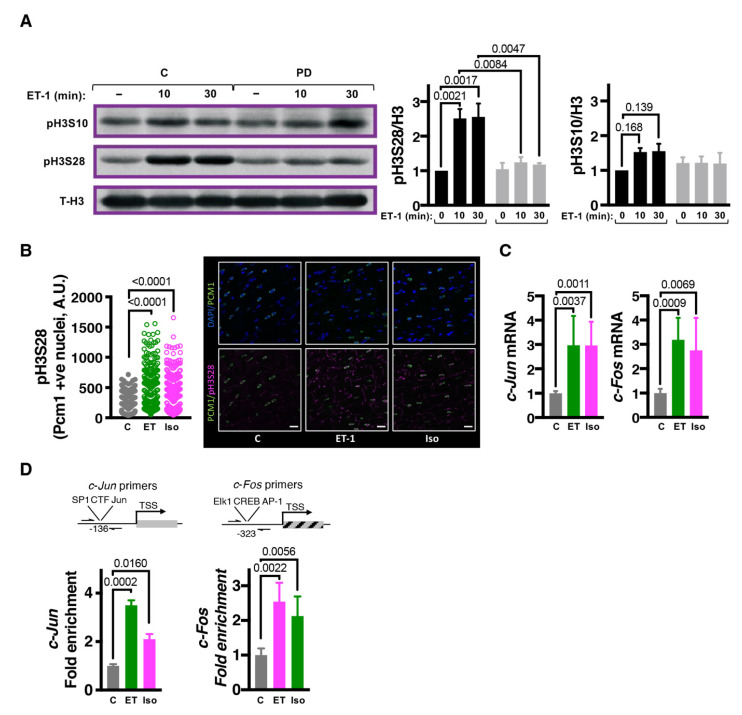
Neurohumoral signalling-induced ERK1/2 activation results in histone H3S28 phosphorylation at IEG promoters. (**A**). Immunoblot analysis of pH3S10 and pH3S28. Left: Representative immunoblots from 1 NRVM preparation probing for pH3S10 and pH3S28 in acid-extracted histones from NRVMs exposed to ET-1 for 0, 10 and 30 min in the presence or absence of PD. Right: Levels of phosphorylated histone normalised to total histone H3. For pH3S10, N = 5. For pH3S28, N = 3. (**B**). Confocal immunofluorescence analysis of pH3S28 in CM in ventricular cardiac sections prepared from rats infused with ET-1 or Iso for 15 min. CM nuclei were demarcated by pericentriolar material 1 (PCM-1; in green) perinuclear staining. Nuclei are stained with DAPI (blue) and pH3S28 in magenta. Scale bar = 25 µm. The plot (**left**) shows quantification of nuclear pH3S28 in PCM-1 positive nuclei. N = 4, 80–125 nuclei per animal. (**C**). RT-qPCR gene expression analysis of IEGs *c-Jun* and *c-Fos* mRNA in left ventricular tissue from Wistar rats administered with ET-1 or Iso through jugular vein infusion and sacrificed 15 min later. N = 7, 8 and 6 for control, ET-1 and Iso infused animals respectively. (**D**). Chromatin immunoprecipitation-qPCR (ChIP-qPCR) analysis of pH3S28 at IEG promoters, *c-Jun* and *c-Fos* in adult male Wistar rats that were administered ET-1 or Iso through jugular vein infusion and sacrificed 15 min later. **Top**: schematic for the site of ChIP primer amplification relative to the transcription start sites. **Below**: quantification of enrichment compared with control (untreated) rats. N = 3.

We first determined whether the nucleosomal response was engaged during the initial phase of the cardiac hypertrophic response to ET-1. To this end, phosphorylation of histone H3 at serines 10 and 28 was quantitated in acid-extracted histones from NRVM exposed to ET-1 for 0, 10 and 30 min. These time points were chosen as they overlapped with the established time-course of *c-Fos* induction, which we have shown previously [11] and here for additional IEG (Figure S1A). The relevance of this model is supported by the hypertrophic response observed when similarly cultured NRVM are harvested after 24 h exposure to ET-1 rather than the time points used for histone phosphorylation analysis (Figure S1B–D). ET-1 promoted a significant increase in pH3S28 at 10 min, which was not additionally increased at the 30 min timepoint (Figure 1A). Notably, the ET-1-stimulated increase in pH3S28 was sensitive to inhibition of ERK1/2 signalling with PD184352 (PD; an inhibitor of the direct upstream kinase of ERK1/2, MEK1/2; Figure S1E) [11,50]. The efficacy of PD184352 inhibition of MAPK signalling was confirmed by its prevention of ET-1-stimulated ERK1/2 phosphorylation (Figure S1F), hypertrophy (Figure S1B–D) and IEG induction (Figure S1G). Phosphorylation of histone H3S10 was not significantly increased following ET-1, and was unaffected by PD (Figure 1A).

**Figure 2:**
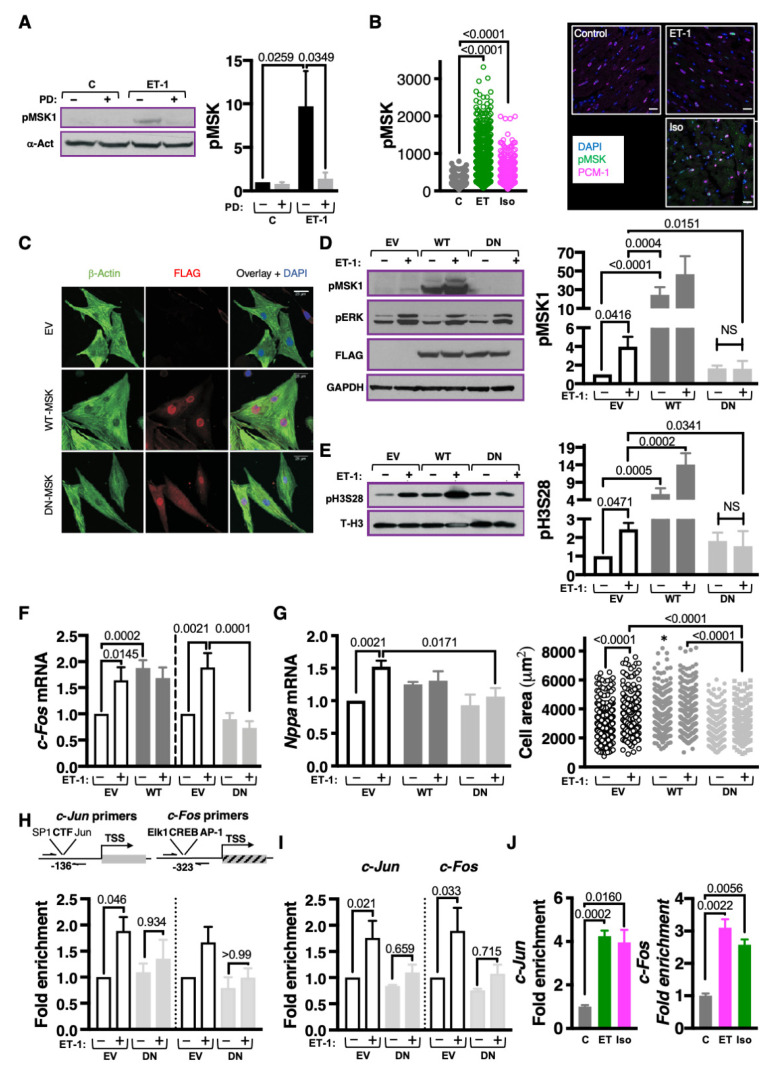
Activated MSK is required for histone H3S28 phosphorylation, recruitment of BRG1 to chromatin and IEG induction in CM. (**A**). Immunoblot showing levels of phosphorylated (activated) MSK in NRVMs ± PD and stimulated ± ET-1 for 10 min. pMSK is normalised to α-Actinin (α-Act) as a loading control. Left: Representative immunoblot. Right: Quantification of pMSK relative to control-vehicle-treated cells. N = 5. (**B**). Confocal immunofluorescence analysis of pMSK in CM in ventricular cardiac sections prepared from rats infused with ET-1 or Iso for 15 min. CM nuclei were demarcated by pericentriolar material 1 (PCM-1; in magenta) perinuclear staining. Nuclei are stained with DAPI (blue) and pMSK in green. Left: Quantification of nuclear pMSK in PCM-1-positive nuclei. N = 4, 200–400 CM nuclei per sample. Right: Confocal images of heart sections from animals treated as indicated. Scale bar = 20 µm. (**C**). Representative confocal images of immunostained NRVMs showing expression of FLAG-tagged WT-MSK and DN-MSK adenoviruses (AdV). Nuclei are stained with DAPI (blue), Beta-Actin in green and FLAG-tagged MSK in red. (**D**). Immunoblotting for pMSK, pERK and FLAG-tagged MSK AdV in NRVMs infected with either empty vector (EV), WT-MSK1 AdV or DN-MSK1 AdV and treated ± 15 min with ET-1, normalised to GAPDH as a loading control. **Left**: Representative immunoblot. **Right**: Quantification of immunoblot, relative to EV. N = 5. (**E**). Immunoblotting for phosphorylated histone H3S28 in NRVMs infected with either empty vector (EV), WT-MSK1 AdV or DN-MSK1 AdV treated ± 15 min with ET-1, normalised to total histone H3 (T-H3) as a loading control. **Left**: Representative immunoblot. **Right**: Quantification of immunoblot data. N = 6. (**F**). Effect of DN-MSK expression on *c-Fos* expression in NRVMs treated with ET-1 for 10 min. *c-Fos* expression was determined by RT-qPCR. Data are presented relative to empty vector. For WT-MSK data (**left**), EV ctrl and WT-MSK ctrl, N = 10, EV ET-1 and WT-MSK ET-1, N = 6. For DN-MSK data (**right**), N = 6. (**G**). Analysis of hypertrophic responses in NRVMs infected with EV or DN-MSK1 AdV treated ± ET-1 for 24 h. **Left**: RT-qPCR expression analysis of *Nppa*/*Anf* mRNA in NRVMs. Data are presented relative to EV untreated cells. For EV ctrl, EV ET-1, WT-MSK ctrl and WT-MSK ET-1, N = 8. For DN-MSK ctrl and DN-MSK ET-1, N = 6. **Right**: Cell area (μm^2^) as a measure of hypertrophy in NRVMs. N = 4, 50–80 cells per condition. * indicates significantly different from EV transduced NRVM not treated with ET-1; p < 0.0001 (**H**). ChIP-qPCR analysis for pH3S28 abundance at *c-Jun* (**left**) and *c-Fos* gene promoter regions in NRVMs infected with EV or DN-MSK1 AdV ± ET-1 for 10 min. **Top**: schematic for the site of ChIP primer amplification relative to the transcription start sites. Below: quantification of enrichment compared with EV AdV untreated NRVMs. For *c-Jun* ChIP data (**Left**), N = 4. For *c-Fos* ChIP data (**Right**), N = 3. (**I**). ChIP-qPCR analysis for BRG1 enrichment at *c-Jun* (left) and *c-Fos* gene promoter regions in NRVMs infected with EV or DN-MSK1 AdV ± ET-1 for 10 min. Quantification of BRG1 enrichment at the *c-Jun* (**Left**) and *c-Fos* (**Right**) promoters compared with EV AdV untreated NRVMs. For *c-Jun* ChIP data (**Left**), N = 4. For *c-Fos* ChIP data (**Right**), N = 3. (**J**). ChIP-qPCR for BRG1 at the *c-Jun* and *c-Fos* gene promoters in left ventricular tissue from adult male Wistar rats that were administered ET-1 or Iso through jugular vein administration and sacrificed 15 min later. N = 3.

To validate these findings in vivo, CM pH3S28 levels were next determined in adult rats following in vivo administration of hypertrophic agonists [11,51]. For these experiments, animals were infused for 15 min via the jugular vein with hypertrophic agonists. In particular, rats were infused with either subpressor levels of ET-1, previously shown to stimulate induction of hypertrophic gene expression, or with the synthetic β-adrenergic agonist isoproterenol (Iso), which is widely used under chronic conditions to evoke a pathological hypertrophic response and heart failure involving CM hypertrophy [5,52,53]. To selectively quantitate pH3S28 in CMs and not in other cardiac cell types, which are in the majority in the heart, pH3S28 was measured by confocal imaging of immunostained heart sections. CM nuclei were identified by their staining for PCM-1. Both ET-1 and Iso infusion significantly increased CM pH3S28 (Figure 1B). mRNA expression of IEGs was also induced following 15 min stimulation in ET-1 and Iso-infused hearts (Figure 2C and Figure S2A). Supporting the pro-hypertrophic effect of these infusions with ET-1 or Iso, *Nppa/Anf* and *Nppb/Bnp* were also induced in these animals (Figure S2B).

To probe whether pH3S28 was increased at IEG promoters following exposure to ET-1 or Iso infusion, chromatin immunoprecipitation (ChIP) experiments were performed. ChIPs were performed using antibodies against pH3S28 and the product detected by qPCR using primers targeting the c-*Jun* and c-*Fos* promoters, as shown in the cartoons (Figure 1D). Consistent with the increased pH3S28 detected by immunoblotting and immunofluorescence analysis, ChIP enrichment for pH3S28-associated IEG promoters was substantially increased following 15 min ET-1 or Iso infusion (Figure 1D).

### 3.2. MSK1/2 Is Activated Following ET-1 Stimulation in an ERK1/2 Dependent Manner

Given its role in phosphorylation of histone H3S28, albeit in non-cardiac tissues, we tested the involvement of MSK1/2 in the phosphorylation of histone H3S28 by ET-1 in NRVM [33,34]. The requirement for ERK1/2 activity for coupling ET-1 stimulation and MSK activation was also tested. MSK1 phosphorylation at S376 (pMSK), a phosphorylation site required for activity, was analysed by immunoblotting of lysates prepared from NRVM stimulated with ET-1, with and without PD (Figure 2A). As shown in the immunoblot and densitometric analysis, ET-1 stimulated an increase in pMSK that was sensitive to MEK/ERK pathway inhibition (Figure 2A). Showing in vivo preservation of this pathway, pMSK levels were also increased in PCM-1+ve CM nuclei in heart sections from rats infused with ET-1 or Iso for 15 min (Figure 2B). Together, these experiments indicate that hypertrophic agonists stimulate a rapid phosphorylation of MSK in CM that is dependent on MEK/ERK signalling.

The requirement for MSK for the ET-1-stimulated phosphorylation of histone H3S28 was next investigated. Owing to the lack of specific pharmacological inhibitors of MSK1/2 [39,41], a molecular approach was used to manipulate its activity. MSK1 signalling was enhanced through overexpression of wild-type MSK1 (WT-MSK), whilst endogenous MSK1 was inhibited by expression of a D565A kinase dead mutant of MSK1 that acts in a dominant–negative fashion (DN-MSK1) [36]. The shared regulatory mechanisms of MSK1 and MSK2 render them equally sensitive to DN-MSK1 inhibition, thereby overcoming possible redundancy [36,54]. Immunofluorescence analysis demonstrated nuclear localisation of adenovirally expressed FLAG tagged WT- and DN-MSK1 in NRVMs (Figure 2C) and immunoblotting showed overexpression of the WT and DN-MSK1 proteins at equivalent levels (detected using anti-FLAG antibody; Figure 2D). Overexpression of WT-MSK1 produced an increase in baseline MSK1 phosphorylation, whereas consistent with its reported dominant negative mode of action, DN-MSK1 expression prevented ET-1-stimulated activation of endogenous MSK1 (Figure 2D). Neither overexpression of WT-MSK1 or DN-MSK1 affected ERK1/2 activation following ET-1 stimulation, indicating that effects of these strategies to modify MSK activity are not mediated via altered ERK1/2 activity but by MSK1 itself (Figure 2D). The consequences of WT and DN-MSK1 expression on ET-1 stimulated histone H3S28 phosphorylation were next measured. Notably, both ET-1 application and WT-MSK1 overexpression increased pH3S28 levels in NRVMs. ET-1 stimulated in pH3S28 was, however, inhibited by DN-MSK1 expression (Figure 2E). No additional effect of ET-1 on pH3S28 was observed in NRVM overexpressing WT-MSK. Together, these data are consistent with MSK being responsible for the phosphorylation of histone H3S28 in ET-1 stimulated CMs.

### 3.3. MSK1/2 Stimulates IEG Induction, Phosphorylation of H3S28 at IEG Promoters and Promotes Induction of Hypertrophic Gene Expression

The requirement for MSK activity for IEG induction and hypertrophic responses was examined (Figure 2F,G). WT-MSK overexpression resulted in a significant elevation in *c-Fos* expression compared to control, which was not additionally increased by 10 min exposure to ET-1 (Figure 2F). Consistent with its effects on kinase activation and histone H3S28 phosphorylation, DN-MSK1 expression significantly inhibited ET-1 stimulated *c-Fos* induction. WT-MSK expression also had a significant effect on cell hypertrophy, stimulating an increase in cell area in the absence of ET-1 (Figure 2G). WT-MSK1 did not promote a significant increase in *Nppa* mRNA above its already increased baseline levels. Notably DN-MSK1 expression suppressed NRVM hypertrophic responses to ET-1, with a significant inhibition of the ET-1 stimulated increases in *Nppa* mRNA and cell size observed (Figure 2G). In line with these findings, dominant negative Jun (DN-Jun) expression inhibited ET-1 induced increases in *Nppa* expression and cell size (Figure S3A,B). Further supporting the relevance of this pathway, ET-1-stimulated increases in histone H3S28 phosphorylation and *c-Fos* and *Nppa* mRNA expression were also abrogated by DN-MSK in ventricular CM from adult rat (Figure S3C,D).

To further examine the relationship between MSK, pH3S28 and IEG induction, we next probed whether MSK was required for the phosphorylation of histone H3S28 at IEG promoters in response to ET-1. ChIP experiments were carried out in NRVMs expressing DN-MSK1 or empty vector control that were exposed to ET-1 or vehicle for 10 min. Since the aim of these experiments was to test the requirement for MSK for the phosphorylation of H3S28 downstream of ET-1 stimulation, the effects of WT-MSK were not examined. In these experiments, the ET-1-stimulated ChIP enrichment of c-*Jun* and c-*Fos* promoters was lost in NRVM overexpressing DN-MSK1 (Figure 2H).

To complement experiments using DN-MSK to suppress MSK activation, an siRNA approach was used to knock down MSK expression. siRNA transfection resulted in ~60% reduction in *Msk1* mRNA compared to NRVM transfected with scrambled control siRNA (Figure S3E). In MSK1 siRNA knockdown (siMsk1) NRVMs, ET-1-stimulated induction of *c-Fos* was significantly blunted after 10 min exposure (Figure S3F). Moreover, siMsk1 prevented ET-1-stimulated hypertrophic gene expression (Figure S3G).

Together, these findings identify MSK1 as the kinase responsible for the phosphorylation of histone H3S28 in IEG promoters and induction of *c-Fos* expression in CM stimulated with ET-1. Moreover, our data show that these events are required for the CM hypertrophic response.

### 3.4. MSK1-Mediated Phosphorylation of H3S28 Recruits BRG1, a Component of the BAF60 Chromatin Remodelling Complex to IEG Loci

IEG induction and pathological cardiac remodelling involves the action of Brahma-related gene-1(BRG1; encoded by gene *SMARCA4*), a component of the BAF (BRG1/Brahma (BRM)-associated factor 60; BAF60) ATP-dependent chromatin remodelling complex [55]. While a relationship between histone H3S28 phosphorylation and BRG1 recruitment has been shown in certain tissue contexts [30], this relationship has not been shown in the cardiac hypertrophic response. Prior to testing the role of BRG1 in IEG induction in CM, its influence on hypertrophic remodelling was first validated. siRNA knockdown of BRG1 expression prevented isoform switching between *Myh6* and *Myh7*, associated with induction of pathological hypertrophy (Figure S3H,I). Notably, BRG1 knockdown also abrogated *c-Fos* induction in ET-1-stimulated NRVMs (Figure S3J). The requirement for pH3S28 for BRG1 occupancy at IEG promoters in NRVM responses to ET-1 was investigated. ChIP experiments were performed on NRVMs expressing DN-MSK1 ± ET-1, as in Figure 2H but using an anti BRG1 antibody. Notably, ET-1 stimulation of NRVMs led to an increase in BRG1 association with the *c-Jun* and *c-Fos* promoters, which was prevented by DN-MSK expression (Figure 2I). Further supporting these in vitro data, ChIP experiments demonstrated enrichment of BRG1 at the *c-Jun* and *c-Fos* promoters in chromatin prepared from hearts from Wistar rats infused with ET-1 or Iso for 15 min (Figure 2J).

Collectively, these data demonstrate that MSK-mediated phosphorylation of histone H3S28 at IEG promoters is a necessary event for the recruitment of the chromatin remodelling complex required for IEG expression and hypertrophic responses to GPCR stimulation.

### 3.5. MSK1/2 Expression Is Required for the Hypertrophic Response In Vivo

The data above indicate that IEG induction in response to neurohormonal stimuli requires MSK phosphorylation of histone H3S28 at IEG loci leading to recruitment of BRG1. In vitro data also show that this pathway is required for the CM hypertrophic response. The requirement for these actions of MSK1/2 during the early phases of the response to hypertrophic stimulation for the later development of hypertrophy in vivo was next examined. To these ends, the effects of mini-pump infusion of Iso were compared between wild type (WT) mice and mice in which both alleles of *Msk* had been deleted (*Msk1/2*^−/−/−/−^; *Msk1/2* knockout (*Msk1/2* KO)) [43]. We focused on the first two weeks of the Iso response as we were interested in the early stages of cardiac remodelling and not in heart failure [52,56].

**Figure 3:**
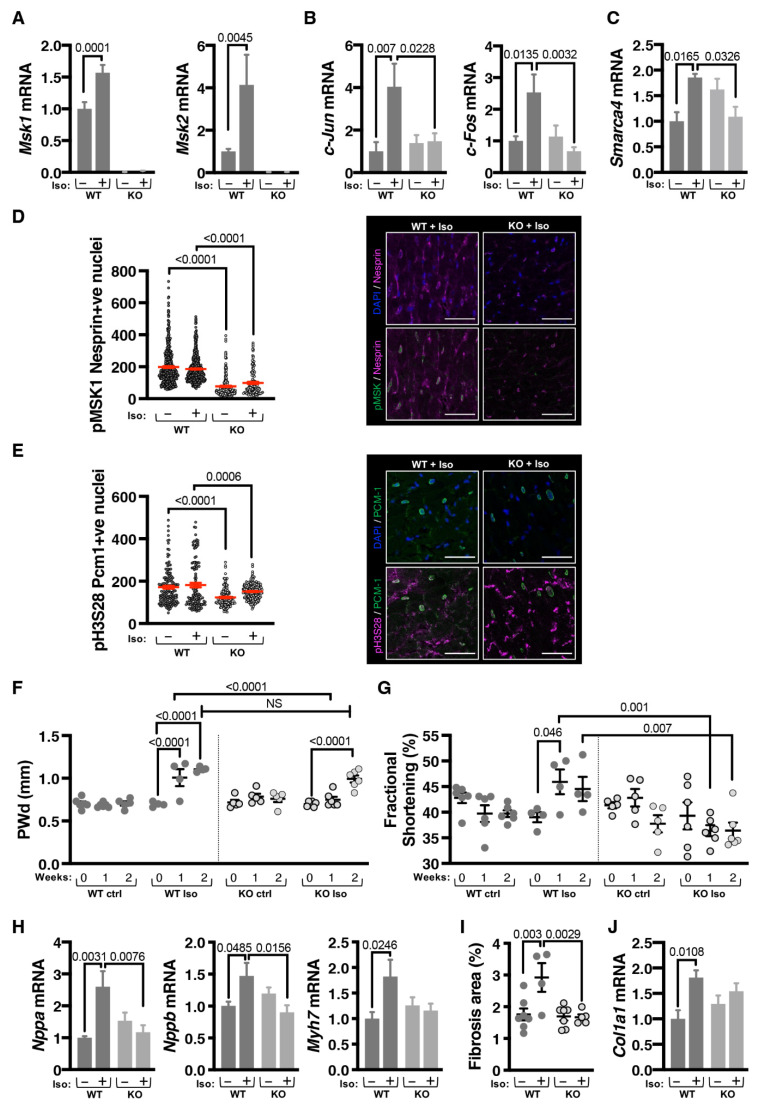
IEG activation and cardiomyocyte hypertrophy is suppressed in vivo in MSK1/2 KO mice. (**A**). RT-qPCR analysis of *Msk1* (**Left**) and *Msk2* (**Right**) mRNA expression in left ventricle from *Msk1/2* KO mice and wild type littermates ± Iso infusion for 2 weeks. WT ctrl, N = 7, WT Iso, N = 4, KO ctrl, N = 5, KO Iso, N = 8. (**B**). RT-qPCR analysis of expression of IEGs *c-Jun* (**Left**) and *c-Fos* (**Right**) in left ventricle from *Msk1/2* KO mice and wild type littermates ± Iso infusion for 2 weeks. WT ctrl, N = 5, WT Iso, N = 4, KO ctrl, N = 5, KO Iso, N = 5. (**C**). RT-qPCR analysis of *Smarca4/Brg1* mRNA expression in left ventricle from *Msk1/2* KO mice and wild type littermates ± Iso infusion for 2 weeks. WT ctrl, N = 5, WT Iso, N = 4, KO ctrl, N = 5, KO Iso, N = 5. (**D**). Immunostaining for pMSK in CM nuclei in left ventricular cardiac sections in *Msk1/2* KO mice and wild type littermates ± Iso infusion for 2 weeks. CM nuclei are demarcated with Nesprin. **Left**: Quantification of pMSK in Nesprin+ve nuclei. **Right**: Representative immunostaining images for pMSK (green), Nesprin (red) and nuclei are stained with DAPI (blue). N = 4, 30–160 nuclei per sample. Scale bar = 50 µm. (**E**). Immunostaining for pH3S28 in CM nuclei in left ventricular cardiac sections in *Msk1/2* KO mice and wild type littermates ± Iso infusion for 2 weeks. CM nuclei are demarcated with PCM-1. **Left**: Quantification of pH3S28 in PCM-1+ve nuclei. **Right**: Representative immunostaining images for pH3S28 (red), PCM-1 (green) and nuclei are stained with DAPI (blue). N = 4, 30–160 nuclei per sample. Scale bar = 50 µm. (**F**). Posterior wall dimension in diastole in *Msk1/2* KO mice and wild type littermates at 1 and 2 weeks ± Iso infusion, derived from 2D echocardiography data. WT ctrl, N = 6, WT Iso, N = 4, KO ctrl, N = 5, KO Iso, N = 6. (**G**). Fractional shortening in *Msk1/2* KO mice and wild type littermates at baseline (Iso = 0) and ± Iso infusion for 1 and 2 weeks (Iso = 1), derived from 2D echocardiography data. WT ctrl, N = 6, WT Iso, N = 4, KO ctrl, N = 5, KO Iso, N = 6. (**H**). RT-qPCR analysis of the markers of pathological hypertrophy *Nppa*/Anf, *Nppb*/Bnp and *Myh*7 mRNA expression in left ventricle from *Msk1/2* KO mice and wild type littermates ± Iso infusion for 2 weeks. N = 5, WT Iso, N = 4, KO ctrl, N = 5, KO Iso, N = 5. (**I**). Quantification of left ventricular interstitial fibrosis, measured as percentage (%) area of extracellular matrix from Picro Sirius Red staining in left ventricular tissue from *Msk1/2* KO mice and wild type littermates ± Iso infusion for 2 weeks. WT ctrl, N = 7, WT Iso, N = 4, KO ctrl, N = 7, KO Iso, N = 5. (**J**). RT-qPCR analysis of *Col1a1* mRNA expression in left ventricular tissue from *Msk1/2* KO mice and wild type littermates ± Iso infusion for 2 weeks. WT ctrl, N = 5, WT Iso, N = 4, KO ctrl, N = 5, KO Iso, N = 5.

The activation of the MSK/pH3S28/BRG1/IEG pathway axis during the Iso infusion and how it was affected by loss of *Msk1/2* was first assessed. As expected, *Msk1* and *Msk2* transcripts were absent in heart tissue of the *Msk1/2* KO mouse (Figure 3A). *Msk1* and *Msk2* mRNA levels were also measured in the Iso-infused WT mice and were found to be significantly upregulated after 2 weeks (Figure 3A). Notably, expression of IEGs including *c-Fos* and *c-Jun* was elevated at 2 weeks in Iso-infused WT mice but not in the *Msk1/2* KO mouse (Figure 3B,C). In line with its role in IEG induction, *Smarca4* (the gene encoding Brg1) expression was also elevated in the Iso-infused WT but not the similarly treated *Msk1/2* KO mouse (Figure 3B,C). Immunofluorescence analysis was carried out to examine levels of nuclear pMSK and pH3S28 (demarcated by PCM-1 or Nesprin perinuclear staining [47]) in CM in sections prepared from Iso-infused WT and *Msk1/2* KO animals. As expected, the signal for pMSK within PCM-1 bounded nuclei was at very low background levels in the *Msk1/2* KO hearts in comparison with control WT hearts. pH3S28 immunostaining was detected in PCM-1 +ve nuclei of WT mice and at a significantly lower level in *Msk1/2* KO (Figure 3D,E). Some non-nuclear staining was observed and considered non-specific since MSK is a nuclear localized kinase. While significantly higher than in *Msk1/2* KO at baseline, no significant effect of Iso on pMSK or pH3S28 was however observed in WT CM. These data are consistent with the absence of MSK protein in the *Msk1/2* KO and the dominant but not exclusive role of MSK as the histone H3S28 kinase.

To determine the wider relevance of the role of MSK in hypertrophic responses, we examined whether increased expression of *Msk1/2*, *Smarca4,* and the IEG they regulate was a feature of other models of hypertrophy. In line with the findings in mice infused with Iso for 2 weeks, expression of IEGs, *Smarca4* and *Msk1/2,* was elevated in CM from rats subjected to constriction of the ascending aorta (AB) for 6 weeks (a model of pathological hypertrophy) [47] (Figure S4A). Indicative of a specific role in pathological cardiac remodelling, no increase in expression of these genes was detected in CM from rats exhibiting a similar degree of hypertrophy induced by 6 weeks treadmill training to elicit physiological hypertrophic remodelling [47] (Figure S3A). *Msk1* and *Msk*2 mRNA levels were also increased in hypertrophic NRVMs after 24 h of ET-1 stimulation (Figure S3B).

Hypertrophic remodelling was next assessed in the *Msk1/2* KO mice. Cardiac function and geometry were measured in vivo by 2D echocardiography prior to the start of the experiment (baseline) and after one and two weeks of Iso infusion. In line with the reported lack of an overt phenotype in *Msk1/2* double KO mice, no differences in cardiac function, indicated by fractional shortening (FS) or posterior wall thickness (PWd) were detected between control WT and *Msk1/2* KO animals at baseline (Figure 3F and Figure S3C). Consistent with the established rapid pro-hypertrophic effect of Iso infusion [52,56], WT mice exhibited an increase in posterior wall thickness after 1 week of Iso that was further increased at 2 weeks. This effect of Iso was significantly delayed in *Msk1/2* KO mice with no increase in PWd detected until 2 weeks of Iso infusion (Figure 3F and Figure S3C). While fractional shortening was significantly increased after 1 week of Iso infusion in WT mice, no such increase was observed in similarly treated *Msk1/2* KO mice, which exhibited a significantly reduced fractional shortening at both 1 and 2 weeks post Iso infusion compared to similarly treated WT mice (Figure 3G).

As a further measure of hypertrophic remodelling, the induction of expression of components of the foetal gene program was assessed by RT-qPCR. While expression of *Nppa*, *Nppb* and *Myh7* was increased in WT mice after Iso infusion, no such effects of Iso were detected in *Msk1/2* KO mice (Figure 3H).

Fibrosis is a common feature of pathological hypertrophy in vivo, including when induced by Iso [5,52]. Histological analysis of left ventricular tissue sections revealed a significant increase in interstitial fibrosis following Iso infusion in WT mice but not in the *Msk1/2* KO mice (Figure 3I and Figure S3D). Supporting the histological analysis, mRNA expression of the extracellular matrix component *Col1a1* was substantially increased in Iso-infused WT mice but not in similarly treated *Msk1/2* KO mice (Figure 3J).

Given the association between CM viability and MAPK pathways, we assessed whether the reduction in fibrosis was associated with reduced cell death, which was contributing to the protective effects of *Msk1/2* KO. TUNEL staining of heart sections revealed a significant Iso-dependent increase in cell death in WT mouse hearts that was not observed in *Msk1/2* KO mice (Figure S3E). Baseline cell death was similar between WT and *Msk1/2* KO mouse hearts. As a further measure of cell death induction, mRNA levels of anti- and pro-apoptotic mediators were analysed (Figure S3F,G). mRNA expression of executioner caspases 3 and 9 (*Casp3* and *Casp9*) of apoptosis and of *Bax*, a pro-apoptotic BH3-only family member, were increased following Iso in WT mice but not in the *Msk1/2* KO mouse (Figure S3F). Conversely, the mRNA encoding anti-apoptotic Bcl-2 was increased in the *Msk1/2* KO mouse following Iso infusion but not in the similarly treated WT mouse (Figure S3G).

Together, these data reveal that MSK activity is required for the induction of hypertrophy in vivo, and that in its absence, hearts are protected from pathological insult.

### 3.6. The MSK1/2/pH3S28/BRG1/IEG Axis Is Engaged in Human Hypertrophic Remodelling

**Figure 4:**
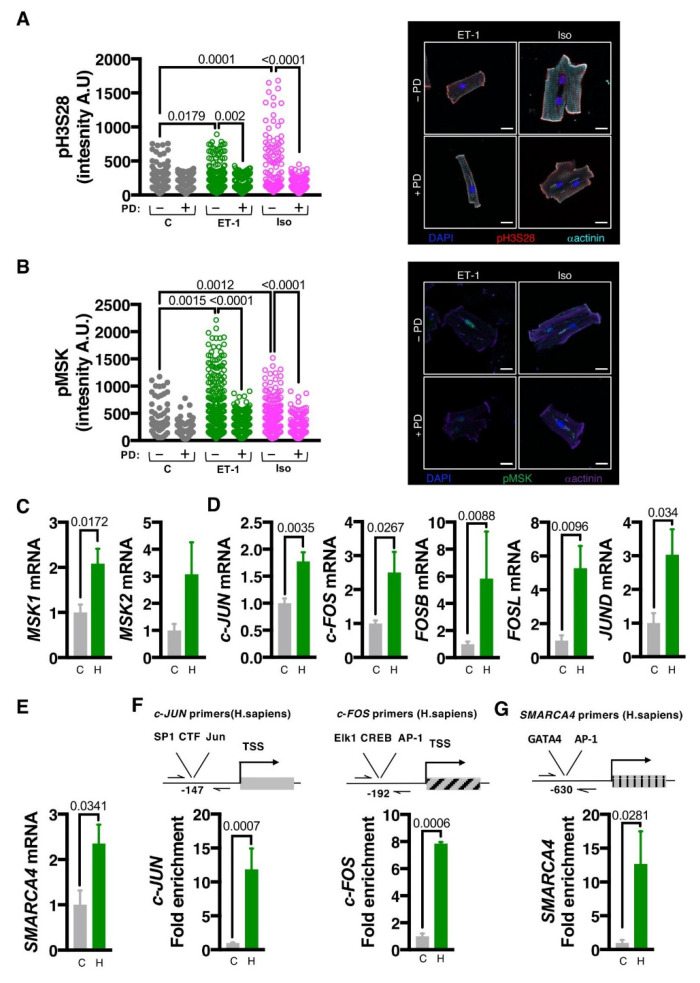
The MAPK-MSK-pH3S28 axis is conserved in the hypertrophic response in humans. (**A**). Confocal immunofluorescence analysis of pH3S28 in isolated human donor CM treated for 15 min with ET-1 or Iso ± PD. **Left**: Quantification of immunostaining of nuclear pH3S28. **Right**: Representative images of isolated CM stained for pH3S28 (red), α-Act (cyan), nuclei stained with DAPI (blue). N = 4, 12–123 cells per sample. Scale bar = 20 µm. (**B**). Confocal immunofluorescence analysis of pMSK in isolated immunostaining in isolated human donor CM, treated for 15 min with ET-1 or Iso ± PD. **Left**: Quantification of immunostaining of nuclear pMSK. **Right**: Representative images of isolated CM stained for pMSK (green), α-Act (purple), nuclei stained with DAPI (blue). N = 4, 14–124 cells per sample. Scale bar = 20 µm. (**C**–**E**). RT-qPCR analysis of mRNA expression of indicated genes in human hypertrophic left ventricular tissue (H) compared with non-failing (C). In Figure 5C,E, C, N = 5, H, N = 4. In Figure 5D, C, N = 4, H, N = 4. (**C**). RT-qPCR analysis of *MSK1* and *MSK2* mRNA expression. (**D**). RT-qPCR analysis of expression of immediate early gene components of the AP-1 transcription factor complex. (**E**). RT-qPCR analysis of *SMARCA4* (*BRG-1*). (**F**). ChIP-qPCR analysis for pH3S28 enrichment at the *c-JUN* and *c-FOS* promoters in PCM-1 +ve CM nuclei from human hypertrophic left ventricular tissue (H) compared with non-failing (C). N = 3. (**G**). ChIP-qPCR for pH3S28 enrichment at the *SMARCA4* promoter in PCM-1 +ve CM nuclei from human hypertrophic left ventricular tissue (H) compared with non-failing (C). N = 3.

We next examined whether the MSK1/2/pH3S28/IEG pathway was conserved in the responses of human CM to hypertrophic agonists. To this end, MSK activation and histone H3S28 phosphorylation following ET-1 and Iso application were analysed in acutely isolated ventricular CM from explanted non-failing donor hearts. The involvement of MAPK activity was also tested. ET-1 and Iso stimulation of human CM resulted in a significant increase in pMSK and pH3S28 (Figure 4A,B). Consistent with our findings in rat ventricular CM, these phosphorylation events were abrogated by ERK1/2 pathway inhibition with PD.

Having shown the persistence of the role of the MSK/pH3S28/IEG axis in the in vivo mouse model of cardiac remodelling and the activation of this pathway by hypertrophic agonists in isolated human CM, we next examined whether this signalling axis remained active in human heart failure. To this end, the expression of MSK1/2, IEGs and early response gene target *SMARCA4* was compared between CM nuclei purified from healthy and hypertrophic human hearts. As in rodents, expression of MSK1/2, IEG components of the AP-1 complex and SMARCA4 were substantially upregulated in heart failure CM (Figure 4C–E). We next analysed pH3S28 in the promoters of *c-FOS*, *C-JUN* and *SMARCA4* by ChIP. Notably, pH3S28 enrichment was observed at all three promoters in CM nuclei isolated from failing compared with healthy control hearts (Figure 4F,G).

**Figure 5:**
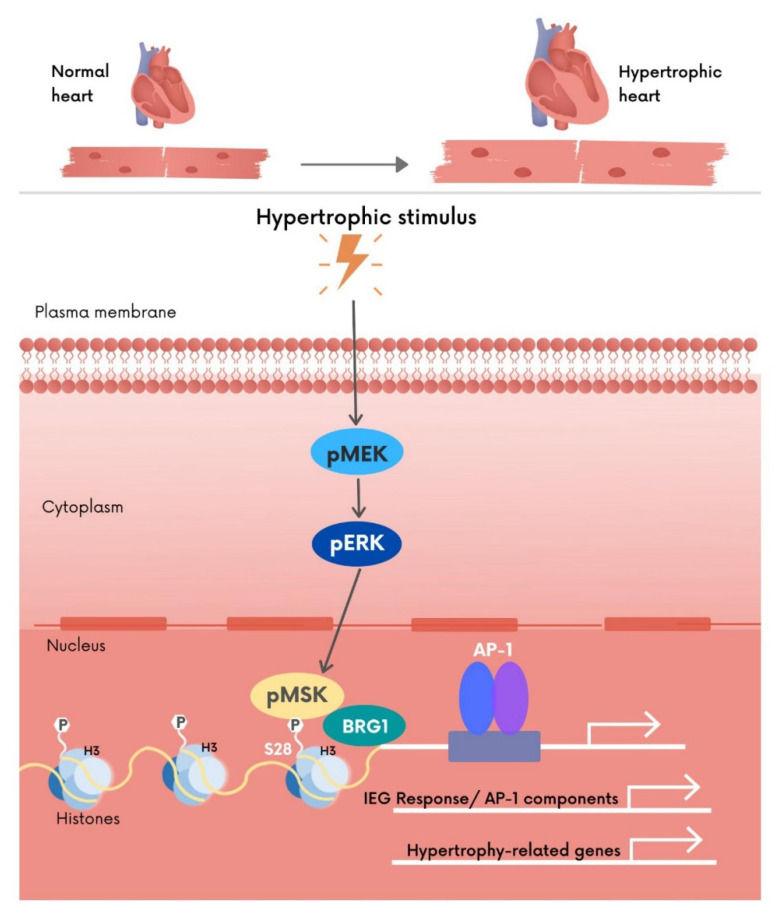
Graphical abstract of main findings of this study indicating pathway by which MSK couples GPCR activation with IEG induction during the cardiac hypertrophic response.

Together, these data show conservation of the MSK pathway to IEG induction in human hearts and support our hypothesis that the MSK/pH3S28/IEG axis is necessary to bring about the initial stages of the CM pathological hypertrophic response (Figure 5).

## 4. Discussion

MAPK pathway regulation of the expression and activity of IEGs is the key to stress-mediated induction of cardiac hypertrophic responses. Here we identified MSK1/2, a kinase-activated downstream of ERK1/2, as being necessary for the initiation of IEG expression in response to pathological hypertrophic cues. MSK1/2 elicited this response through phosphorylation of histone H3S28 allowing recruitment of the ATP-dependent chromatin remodeller, BRG1. In the absence of this response, gene expression changes and tissue remodelling associated with cardiac hypertrophy was attenuated. Notably, live-cell functional assays and analysis of post-mortem human hypertrophic hearts revealed conservation of this mechanism in humans. These data are summarised in the cartoon in Figure 5.

MSK1/2-mediated phosphorylation of histone H3S10 and H3S28 transduces MAPK activation in response to mitogenic stimulation activation to induction of IEG expression in a wide range of tissues [32,33,55,57]. Until now, a mechanism involving MSKs acting as histone kinases contributing to the induction of IEG and of hypertrophic gene expression via a nucleosomal response has not been demonstrated in CM. MSKs have been reported to be activated during hypertrophy, however, and a requirement for MSK activity for the induction of hypertrophy of isolated CM in vitro was previously invoked [37,38,40]. The poor selectivity of the pharmacological inhibitors of MSK used, and the exclusive use of isolated cells in these studies, cast doubt on the conclusions drawn. Indeed, the inhibitors used show equivalent or greater activity on a number of other protein kinases including PKCs, RSK and S6 kinase, with known roles in cardiac hypertrophy [9,39,41]. Moreover, Ro318220 used in the aforementioned studies to inhibit MSK has since been described as an activator of c-Jun N-terminal kinase (which lies upstream of IEG induction) and glycogen synthase [58]. Our data identify MSKs as a downstream target of the ERK MAPK pathway in CM, contributing to its stimulation of IEG expression. The data presented in this article are the first to show a bona fide role for MSK in cardiac transcriptional and hypertrophic responses and to describe its potential mechanism of action. These conclusions are strengthened by the specific molecular tools and animal models used to ensure the specific manipulation of MSK activity required to test its requirement in signalling pathways and hypertrophy induction. The data presented here demonstrate, for the first time, phosphorylation of H3S28 by MSK in CM following exposure to pathological stressors associated with hypertrophy induction. Further, this phosphorylation event is required for IEG activation and CM hypertrophy. In contrast to previous in vitro studies [11,37,38], we also identify the contribution of MSK1/2 and its mechanism of action to IEG induction and hypertrophic responses in vivo. While in vitro hypertrophic responses were completely abrogated, the in vivo hypertrophic response was not but was rather delayed, suggesting activation of redundant or compensatory pathways. Further, the specific role for MSK1/2 in responding to stress stimuli is consistent with the lack of overt phenotype in *Msk1/2* double KO mice [43]. Importantly, the pathway from GPCR stimulation, via MSK activation and phosphorylation of histone H3S28 on IEG loci was conserved to human. Not only could we show increased activation of this pathway in explanted failing hearts, we could also demonstrate in CM isolated from human hearts acute activation of this pathway by disease-relevant neurohormonal stimuli. The continued activity of this signaling axis in human and mouse heart failure described here is perhaps surprising given the transient increase in AP-1 factors following application of stressors reported [11,24,25,26,59], but may point to their continued role in hypertrophic transcriptional responses beyond the initial induction of IEG.

The role of MSK in hypertrophic remodelling that we report shows some overlaps with that previously described for its upstream kinases of the ERK MAPK pathway and for other MAPK pathways that converge on AP-1 factor activation [5,25,26,60]. Of particular note, like MSKs, many of these kinases are required for the initial adaptive responses to hypertrophic stimuli in vivo. Moreover, overexpression of MSK, as shown for many MAPK pathways, is also sufficient to induce cardiac hypertrophy [61]. In NRVM overexpressing MSK, however, no additional effect of hypertrophic stimulation with ET-1 was observed. This is perhaps not surprising given the substantially greater phosphorylation of histone H3S28 in MSK overexpressing NRVM than in NRVM exposed to ET-1 alone. These data further support the pivotal nature of MSK in coupling the effects of hypertrophic stimulation with histone H3S28 phosphorylation, IEG induction and hypertrophic responses. Further supporting a linear relationship between GPCR stimulation, MEK/ERK and MSK signalling, MSK activation, histone H3S28 phosphorylation and downstream transcriptional and hypertrophic responses were dependent on MEK/ERK activity. Contrasting with the effects of knockout of certain MAPK or AP-1 factors [25,26], however, CM death and fibrosis were not exacerbated in the *Msk1/2* KO mouse following Iso infusion. These data likely reflect the complex nature of MAPK and AP-1 factor signaling, which is highly sensitive to expression levels, activity and stoichiometry/complement of participating members [16,17]. For example, whereas in vivo overexpression of ERKs is not sufficient to induce cardiac hypertrophy, overexpression of its direct upstream kinase MEK induces a concentric hypertrophic response [61]. Induction of ERK1/2 expression following overexpression of the small GTPase protein Ras, which lies between GPCR activation and ERK, results in cardiomyopathy, however [62]. Notably, loss of ERK2, which represents 50–70% of ERK activity in the heart, attenuates the initial compensatory phase of the hypertrophic response and causes a direct progression to a cardiomyopathic phenotype associated with substantial CM death [60]. Surprisingly, conditional deletion of both ERK alleles does not prevent pathological hypertrophic growth [63]. ERK1/2 likely makes different contributions to the different forms of hypertrophy—while ERK1/2 mediates concentric growth responses to stimulus, it prevents eccentric growth [7,63,64]. As we suggest for MSKs, ERK2 is not required for physiological cardiac remodelling in response to 4 weeks of swim training, indicating independent pathways for adaptive hypertrophy in response to pathological or physiological stimuli [60]. A similar requirement for the acute adaptive hypertrophic growth and repression of maladaptive growth is reported for c-Jun NH_2_ terminal kinase (JNK1) [65]. Through deletion of this kinase, mice exhibit a loss of adaptive hypertrophic responses and subsequent direct progression to cardiac dilation [65]. Moreover, MKK4, which lies upstream of JNK and p38MAPK, is also required for this hypertrophic response [5]. Consistent with these actions of JNK, c-Jun acts in a protective manner, preventing maladaptive responses to stress [26]. While JunD also protects CM from maladaptive remodelling, its activity is decreased in pathology, leading to a reduction in AP-1 complexes in which it participates, thereby resulting in a greater influence of other AP-1 factors on downstream signalling [24,25]. As a consequence, pathological cardiac remodelling develops. Further contributing to any differences between the effects of loss of MSK activity on IEG signalling compared to knockout of individual AP-1 factors or of relevant upstream kinases are the redundancy and changes in stoichiometry of AP-1 components associated with AP-1 factor KO [17,25]. Specifically, in the absence of MSK activity, the nucleosomal response which majorly participates in IEG induction, including of AP-1 factors, is prevented, thereby eliciting more widespread consequences for their downstream activities [32,33,43].

Promoter histone H3S28 phosphorylation is an important step in the induction of IEG expression. This phosphorylation event results in increased recruitment of BRG1, a component of the BAF60 chromatin remodelling complex, to modified regions of chromatin [55]. Notably, BRG1 plays an important role in chromatin remodelling and transcriptional responses during cardiac development and disease. Indeed, BRG1 recruitment to the *Myh6/7* locus is involved in the switching of these myosin heavy chain isoforms during CM maturation and in response to stress [55]. Suggesting a contribution to the same signalling pathway, BRG1 knockdown resulted in similar consequences to DN-MSK expression, with a loss of induction of *c-Fos* and hypertrophic gene expression in response to ET-1 observed. Moreover, increased BRG1 recruitment to IEG loci following ET-1 stimulation supports these observations. Based on our data, we propose that histone H3S28 phosphorylation by MSK represents an initial step in mediating this hypertrophic response. The recruitment of BRG1 further contributes to modulation of the epigenetic landscape of the heart through recruiting factors including EZH2 to acetylate H3K27 at mesoderm enhancers and for polycomb-mediated repression of non-mesodermal genes [66]. Such a role for MSK-mediated phosphorylation of histone H3S28 is described in neuronal differentiation [66]. H3K27me3 is also lost at activated gene promoters in hypertrophy and disease in CM [47,67]. Whether loss of this mark is associated with gain of pH3S28 is not determined in CM transcriptional responses, although in other contexts, phosphorylation of histone H3S28 displaces the polycomb repressor complex (PRC), allowing H3K27 acetylation [68].

Phosphorylation of histone H3 has previously been described in CM, although CaMKII was reported to be responsible [69,70]. CaMKII was found to directly bind and phosphorylate histone H3S28. In end-stage heart failure as well as in a CaMKII transgenic mouse model, CaMKII-mediated phosphorylation of histone H3S28 in the haemoglobin promoter resulted in enhanced expression in adult CM [70]. Whether this mechanism is protective or contributes to the pathological phenotype is undetermined although in a separate study in Drosophila in which a mutated histone H3S28A mutant was expressed, thereby preventing phosphorylation, improved cardiac function under stress conditions was observed [71]. Notably, CaMKII contributed to a delayed but sustained elevation of global pH3S28 but not to the early peak in pH3S28 observed in response to catecholaminergic stimulation [70]. Our description of a role for MSK in the phosphorylation of histone H3S28 at IEG loci in the minutes following agonist stimulation may suggest a model whereby MSKs mediate the induction of IEGs during hypertrophy whereas CaMKII-dependent phosphorylation of histone H3S28 is involved in the control of the expression of genes involved in later stages of the hypertrophic response. While CaMKII may indeed play a role at certain gene loci, the substantial decrease in histone H3S28 phosphorylation in *Msk1/2* KO animals observed in this study would however suggest that MSKs make a substantial contribution to pH3S28 levels, particularly at IEG loci and during disease remodelling. The absence of an increase in pMSK and pH3S28 levels in the WT mice treated with Iso for 2 weeks is consistent with our previous findings that ERK1/2 signalling activity declines to basal levels within the first days of agonist treatment. Inhibitor studies indicate, however, that despite levels of active kinase being below detection limits, this pathway continues to signal to maintain elevated transcription of its targets [11]. CaMKII is also shown to phosphorylate histone H3S10, leading to the induction of foetal and pro-hypertrophic genes [70,72]. Notably, in the latter study, no CaMKII-dependent phosphorylation of histone H3S28 was detected. The nuclear localisation of this kinase together with its identified role in HDAC phosphorylation provides a mechanism to remodel chromatin in a manner optimal for stimulation of MEF2-dependent gene expression during hypertrophic remodelling [72]. As we did not detect a robust change in pH3S10 in NRVMs exposed to hypertrophic stimuli, we did not examine phosphorylation of this residue in disease in vivo. Together, CaMKII phosphorylation of histones H3S10 and H3S28 and histone H3S28 phosphorylation by MSK represent a mechanism that permits different stimuli at different phases of their action to selectively control the expression of discrete cohorts of target genes.

Other targets of MSK involved in the cardiac hypertrophic response have been described. The first substrate of MSK identified was the cAMP-Responsive Element-Binding Protein (CREB) transcription factor, which binds cAMP response DNA elements (CRE), associating with the histone acetyltransferase CREB-binding protein (CBP/P300) to activate transcription. CREB itself is also phosphorylated by a number of different kinases, including protein kinase A (PKA) [73]. The role of MSK-activated CREB in vivo is controversial. Several studies have demonstrated that PKA- but not MSK-mediated CREB phosphorylation leads to CBP or p300 recruitment [74]. Cardiac-specific expression of a dominant negative form of CREB (DN-CREB) leads to a dilated cardiomyopathy phenotype [75]. Given the extreme phenotype of DN-CREB in contrast to the relatively benign *Msk1/2* double KO, it is likely that normal CREB activity is independent of MSK in the heart.

The lack of a cardiac phenotype of *Msk1/2* KO under baseline conditions suggests a limited role for MSK1/2 in the normal physiological activity of CM. The stress-specific function of MSK may therefore endow it with the necessary qualities of being therapeutically targetable to prevent or ameliorate disease. To date, however, no specific pharmacological inhibitors suitable for in vivo use are available and those currently used in in vitro mechanistic studies show efficacy at multiple other kinases important in cardiac remodelling [39,41]. The development of CRISPR/Cas9 targeted MSK may, however, provide a solution to modify MSK activity at defined loci [76]. Specific inhibitors of kinases upstream of MSK, including members of the ERK/MAPK pathway, may also be used to inhibit its activity, although the baseline and pleiotropic activities of these kinases in the heart make them less ideal targets for manipulation.

## 5. Conclusions

Our data identified MSK as a pivotal and missing link between ERK/MAPK activation, histone H3S28 phosphorylation, IEG induction and CM hypertrophy induction. Further studies will lead to the identification of the wider significance of MSK-induced histone H3S28 phosphorylation in the hypertrophic response and how it may be manipulated for therapeutic benefit.

## Data Availability

Immunoblot data is available. Data is contained within the article or Supplementary Material. RNA-Seq data analysed are available at NCBI’s Gene Expression Omnibus (GEO) database (GEO GSE66653).

## Supplementary Materials

The following supporting information can be downloaded at: https://www.mdpi.com/article/10.3390/cells11040604/s1, Figure S1, Supplementary Figure S1: IEG induction is activated by acute hypertrophic stimulation and attenuated by suppression of ERK signalling in vitro; Figure S2, Histone H3S28 phosphorylation is associated with induction of expression of IEG and hypertrophy-related genes; Figure S3, IEG induction is a conserved feature of hypertrophy in adult CM; Figure S4, Genetic MSK inhibition attenuates IEG activation and cardiomyocyte hypertrophy in vivo. Table S1, Antibody information and dilutions; Table S2, Primer sequence information; Table S3, Human non-failing donor samples for cardiomyocyte isolation; Table S4, Human control and hypertrophic left ventricular cardiomyocyte nuclei. Supplementary File: Supplementary Materials and Methods.

## Abbreviations

Ascending aortic banding, AB; cytosine b-D-arabinofuranoside, ara-C; Histone 3, H3; Histone H3 Serine 10, H3S10; phosphorylated histone H3S10, p-H3S10; Histone H3 Serine 28, H3S28; phosphorylated histone H3S28, pH3S28; immediate early gene, IEG; isoproterenol, Iso; PD184352, PD.

## References

[1] Timmis A., Townsend N., Gale C.P., Torbica A., Lettino M., Petersen S.E., Mossialos E.A., Maggioni A.P., Kazakiewicz D., May H.T. (2020). European Society of Cardiology: Cardiovascular Disease Statistics 2019. Eur. Heart J..

[2] Alkass K., Panula J., Westman M., Wu T.D., Guerquin-Kern J.L., Bergmann O. (2015). No Evidence for Cardiomyocyte Number Expansion in Preadolescent Mice. Cell.

[3] Drawnel F.M., Archer C.R., Roderick H.L. (2013). The role of the paracrine/autocrine mediator endothelin-1 in regulation of cardiac contractility and growth. Br. J. Pharmacol..

[4] Wang J., Gareri C., Rockman H.A. (2018). G-Protein-Coupled Receptors in Heart Disease. Circ. Res..

[5] Liu W., Zi M., Jin J., Prehar S., Oceandy D., Kimura T.E., Lei M., Neyses L., Weston A.H., Cartwright E.J. (2009). Cardiac-specific deletion of mkk4 reveals its role in pathological hypertrophic remodeling but not in physiological cardiac growth. Circ. Res..

[6] Rose B.A., Force T., Wang Y. (2010). Mitogen-activated protein kinase signaling in the heart: Angels versus demons in a heart-breaking tale. Physiol. Rev..

[7] Bueno O.F., De Windt L.J., Tymitz K.M., Witt S.A., Kimball T.R., Klevitsky R., Hewett T.E., Jones S.P., Lefer D.J., Peng C.F. (2000). The MEK1-ERK1/2 signaling pathway promotes compensated cardiac hypertrophy in transgenic mice. EMBO J..

[8] Garrington T.P., Johnson G.L. (1999). Organization and regulation of mitogen-activated protein kinase signaling pathways. Curr. Opin. Cell. Biol..

[9] Heineke J., Molkentin J.D. (2006). Regulation of cardiac hypertrophy by intracellular signalling pathways. Nat. Rev. Mol. Cell. Biol..

[10] Sanna B., Bueno O.F., Dai Y.S., Wilkins B.J., Molkentin J.D. (2005). Direct and indirect interactions between calcineurin-NFAT and MEK1-extracellular signal-regulated kinase 1/2 signaling pathways regulate cardiac gene expression and cellular growth. Mol. Cell. Biol..

[11] Archer C.R., Robinson E.L., Drawnel F.M., Roderick H.L. (2017). Endothelin-1 promotes hypertrophic remodelling of cardiac myocytes by activating sustained signalling and transcription downstream of endothelin type A receptors. Cell. Signal..

[12] Iwaki K., Sukhatme V.P., Shubeita H.E., Chien K.R. (1990). Alpha- and beta-adrenergic stimulation induces distinct patterns of immediate early gene expression in neonatal rat myocardial cells. fos/jun expression is associated with sarcomere assembly; Egr-1 induction is primarily an alpha 1-mediated response. J. Biol. Chem..

[13] Izumo S., Nadal-Ginard B., Mahdavi V. (1988). Protooncogene induction and reprogramming of cardiac gene expression produced by pressure overload. Proc. Natl. Acad. Sci. USA.

[14] Cullingford T.E., Markou T., Fuller S.J., Giraldo A., Pikkarainen S., Zoumpoulidou G., Alsafi A., Ekere C., Kemp T.J., Dennis J.L. (2008). Temporal regulation of expression of immediate early and second phase transcripts by endothelin-1 in cardiomyocytes. Genome Biol..

[15] Gille H., Sharrocks A.D., Shaw P.E. (1992). Phosphorylation of transcription factor p62TCF by MAP kinase stimulates ternary complex formation at c-fos promoter. Nature.

[16] Karin M., Liu Z., Zandi E. (1997). AP-1 function and regulation. Curr. Opin. Cell. Biol..

[17] Hess J., Angel P., Schorpp-Kistner M. (2004). AP-1 subunits: Quarrel and harmony among siblings. J. Cell. Sci..

[18] Eferl R., Wagner E.F. (2003). AP-1: A double-edged sword in tumorigenesis. Nat. Rev. Cancer.

[19] Glover J.N., Harrison S.C. (1995). Crystal structure of the heterodimeric bZIP transcription factor c-Fos-c-Jun bound to DNA. Nature.

[20] Chen L., Glover J.N., Hogan P.G., Rao A., Harrison S.C. (1998). Structure of the DNA-binding domains from NFAT, Fos and Jun bound specifically to DNA. Nature.

[21] Torgerson T.R., Colosia A.D., Donahue J.P., Lin Y.Z., Hawiger J. (1998). Regulation of NF-kappa B, AP-1, NFAT, and STAT1 nuclear import in T lymphocytes by noninvasive delivery of peptide carrying the nuclear localization sequence of NF-kappa B p50. J. Immunol..

[22] Hilfiker-Kleiner D., Hilfiker A., Castellazzi M., Wollert K.C., Trautwein C., Schunkert H., Drexler H. (2006). JunD attenuates phenylephrine-mediated cardiomyocyte hypertrophy by negatively regulating AP-1 transcriptional activity. Cardiovasc. Res..

[23] Omura T., Yoshiyama M., Yoshida K., Nakamura Y., Kim S., Iwao H., Takeuchi K., Yoshikawa J. (2002). Dominant negative mutant of c-Jun inhibits cardiomyocyte hypertrophy induced by endothelin 1 and phenylephrine. Hypertension.

[24] Hilfiker-Kleiner D., Hilfiker A., Kaminski K., Schaefer A., Park J.K., Michel K., Quint A., Yaniv M., Weitzman J.B., Drexler H. (2005). Lack of JunD promotes pressure overload-induced apoptosis, hypertrophic growth, and angiogenesis in the heart. Circulation.

[25] Ricci R., Eriksson U., Oudit G.Y., Eferl R., Akhmedov A., Sumara I., Sumara G., Kassiri Z., David J.P., Bakiri L. (2005). Distinct functions of junD in cardiac hypertrophy and heart failure. Genes Dev..

[26] Windak R., Muller J., Felley A., Akhmedov A., Wagner E.F., Pedrazzini T., Sumara G., Ricci R. (2013). The AP-1 transcription factor c-Jun prevents stress-imposed maladaptive remodeling of the heart. PLoS ONE.

[27] Amirak E., Fuller S.J., Sugden P.H., Clerk A. (2013). p90 ribosomal S6 kinases play a significant role in early gene regulation in the cardiomyocyte response to G(q)-protein-coupled receptor stimuli, endothelin-1 and alpha(1)-adrenergic receptor agonists. Biochem. J..

[28] Clayton A.L., Rose S., Barratt M.J., Mahadevan L.C. (2000). Phosphoacetylation of histone H3 on c-fos- and c-jun-associated nucleosomes upon gene activation. EMBO J..

[29] Dyson M.H., Thomson S., Inagaki M., Goto H., Arthur S.J., Nightingale K., Iborra F.J., Mahadevan L.C. (2005). MAP kinase-mediated phosphorylation of distinct pools of histone H3 at S10 or S28 via mitogen- and stress-activated kinase 1/2. J Cell. Sci..

[30] Drobic B., Perez-Cadahia B., Yu J., Kung S.K., Davie J.R. (2010). Promoter chromatin remodeling of immediate-early genes is mediated through H3 phosphorylation at either serine 28 or 10 by the MSK1 multi-protein complex. Nucleic Acids Res..

[31] Duncan E.A., Anest V., Cogswell P., Baldwin A.S. (2006). The kinases MSK1 and MSK2 are required for epidermal growth factor-induced, but not tumor necrosis factor-induced, histone H3 Ser10 phosphorylation. J. Biol. Chem..

[32] Josefowicz S.Z., Shimada M., Armache A., Li C.H., Miller R.M., Lin S., Yang A., Dill B.D., Molina H., Park H.S. (2016). Chromatin Kinases Act on Transcription Factors and Histone Tails in Regulation of Inducible Transcription. Mol. Cell..

[33] Soloaga A., Thomson S., Wiggin G.R., Rampersaud N., Dyson M.H., Hazzalin C.A., Mahadevan L.C., Arthur J.S. (2003). MSK2 and MSK1 mediate the mitogen- and stress-induced phosphorylation of histone H3 and HMG-14. EMBO J..

[34] Malakhova M., Kurinov I., Liu K., Zheng D., D’Angelo I., Shim J.H., Steinman V., Bode A.M., Dong Z. (2009). Structural diversity of the active N-terminal kinase domain of p90 ribosomal S6 kinase 2. PLoS ONE.

[35] McCoy C.E., Campbell D.G., Deak M., Bloomberg G.B., Arthur J.S. (2005). MSK1 activity is controlled by multiple phosphorylation sites. Biochem. J..

[36] Deak M., Clifton A.D., Lucocq L.M., Alessi D.R. (1998). Mitogen- and stress-activated protein kinase-1 (MSK1) is directly activated by MAPK and SAPK2/p38, and may mediate activation of CREB. EMBO J..

[37] Markou T., Cieslak D., Gaitanaki C., Lazou A. (2009). Differential roles of MAPKs and MSK1 signalling pathways in the regulation of c-Jun during phenylephrine-induced cardiac myocyte hypertrophy. Mol. Cell. Biochem..

[38] Markou T., Hadzopoulou-Cladaras M., Lazou A. (2004). Phenylephrine induces activation of CREB in adult rat cardiac myocytes through MSK1 and PKA signaling pathways. J. Mol. Cell. Cardiol..

[39] Alessi D.R. (1997). The protein kinase C inhibitors Ro 318220 and GF 109203X are equally potent inhibitors of MAPKAP kinase-1beta (Rsk-2) and p70 S6 kinase. FEBS Lett..

[40] Markou T., Lazou A. (2002). Phosphorylation and activation of mitogen- and stress-activated protein kinase-1 in adult rat cardiac myocytes by G-protein-coupled receptor agonists requires both extracellular-signal-regulated kinase and p38 mitogen-activated protein kinase. Biochem. J..

[41] Naqvi S., Macdonald A., McCoy C.E., Darragh J., Reith A.D., Arthur J.S. (2012). Characterization of the cellular action of the MSK inhibitor SB-747651A. Biochem. J..

[42] Arthur J.S., Cohen P. (2000). MSK1 is required for CREB phosphorylation in response to mitogens in mouse embryonic stem cells. FEBS Lett..

[43] Wiggin G.R., Soloaga A., Foster J.M., Murray-Tait V., Cohen P., Arthur J.S. (2002). MSK1 and MSK2 are required for the mitogen- and stress-induced phosphorylation of CREB and ATF1 in fibroblasts. Mol. Cell. Biol..

[44] Higazi D.R., Fearnley C.J., Drawnel F.M., Talasila A., Corps E.M., Ritter O., McDonald F., Mikoshiba K., Bootman M.D., Roderick H.L. (2009). Endothelin-1-stimulated InsP3-induced Ca2+ release is a nexus for hypertrophic signaling in cardiac myocytes. Mol. Cell..

[45] Drawnel F.M., Wachten D., Molkentin J.D., Maillet M., Aronsen J.M., Swift F., Sjaastad I., Liu N., Catalucci D., Mikoshiba K. (2012). Mutual antagonism between IP(3)RII and miRNA-133a regulates calcium signals and cardiac hypertrophy. J. Cell. Biol..

[46] Dries E., Santiago D.J., Gilbert G., Lenaerts I., Vandenberk B., Nagaraju C.K., Johnson D.M., Holemans P., Roderick H.L., Macquaide N. (2018). Hyperactive ryanodine receptors in human heart failure and ischaemic cardiomyopathy reside outside of couplons. Cardiovasc. Res..

[47] Thienpont B., Aronsen J.M., Robinson E.L., Okkenhaug H., Loche E., Ferrini A., Brien P., Alkass K., Tomasso A., Agrawal A. (2017). The H3K9 dimethyltransferases EHMT1/2 protect against pathological cardiac hypertrophy. J. Clin. Invest..

[48] Vandesompele J., De Preter K., Pattyn F., Poppe B., Van Roy N., De Paepe A., Speleman F. (2002). Accurate normalization of real-time quantitative RT-PCR data by geometric averaging of multiple internal control genes. Genome Biol..

[49] Livak K.J., Schmittgen T.D. (2001). Analysis of relative gene expression data using real-time quantitative PCR and the 2(-Delta Delta C(T)) Method. Methods.

[50] Allen L.F., Sebolt-Leopold J., Meyer M.B. (2003). CI-1040 (PD184352), a targeted signal transduction inhibitor of MEK (MAPKK). Semin. Oncol..

[51] Kadel K.A., Heistad D.D., Faraci F.M. (1990). Effects of endothelin on blood vessels of the brain and choroid plexus. Brain Res..

[52] Boluyt M.O., Long X., Eschenhagen T., Mende U., Schmitz W., Crow M.T., Lakatta E.G. (1995). Isoproterenol infusion induces alterations in expression of hypertrophy-associated genes in rat heart. Am. J. Physiol..

[53] Werhahn S.M., Kreusser J.S., Hagenmuller M., Beckendorf J., Diemert N., Hoffmann S., Schultz J.H., Backs J., Dewenter M. (2021). Adaptive versus maladaptive cardiac remodelling in response to sustained beta-adrenergic stimulation in a new ‘ISO on/off model’. PLoS ONE.

[54] Zhong S., Jansen C., She Q.B., Goto H., Inagaki M., Bode A.M., Ma W.Y., Dong Z. (2001). Ultraviolet B-induced phosphorylation of histone H3 at serine 28 is mediated by MSK1. J. Biol. Chem..

[55] Hang C.T., Yang J., Han P., Cheng H.L., Shang C., Ashley E., Zhou B., Chang C.P. (2010). Chromatin regulation by Brg1 underlies heart muscle development and disease. Nature.

[56] Wang J.J., Rau C., Avetisyan R., Ren S., Romay M.C., Stolin G., Gong K.W., Wang Y., Lusis A.J. (2016). Genetic Dissection of Cardiac Remodeling in an Isoproterenol-Induced Heart Failure Mouse Model. PLoS Genet..

[57] Mahadevan L.C., Willis A.C., Barratt M.J. (1991). Rapid histone H3 phosphorylation in response to growth factors, phorbol esters, okadaic acid, and protein synthesis inhibitors. Cell.

[58] Standaert M.L., Bandyopadhyay G., Antwi E.K., Farese R.V. (1999). RO 31-8220 activates c-Jun N-terminal kinase and glycogen synthase in rat adipocytes and L6 myotubes. Comparison to actions of insulin. Endocrinology.

[59] Santalucia T., Christmann M., Yacoub M.H., Brand N.J. (2003). Hypertrophic agonists induce the binding of c-Fos to an AP-1 site in cardiac myocytes: Implications for the expression of GLUT1. Cardiovasc. Res..

[60] Ulm S., Liu W., Zi M., Tsui H., Chowdhury S.K., Endo S., Satoh Y., Prehar S., Wang R., Cartwright E.J. (2014). Targeted deletion of ERK2 in cardiomyocytes attenuates hypertrophic response but provokes pathological stress induced cardiac dysfunction. J. Mol. Cell. Cardiol..

[61] Mutlak M., Kehat I. (2015). Extracellular signal-regulated kinases 1/2 as regulators of cardiac hypertrophy. Front Pharmacol..

[62] Wu G., Yussman M.G., Barrett T.J., Hahn H.S., Osinska H., Hilliard G.M., Wang X., Toyokawa T., Yatani A., Lynch R.A. (2001). Increased myocardial Rab GTPase expression: A consequence and cause of cardiomyopathy. Circ. Res..

[63] Kehat I., Davis J., Tiburcy M., Accornero F., Saba-El-Leil M.K., Maillet M., York A.J., Lorenz J.N., Zimmermann W.H., Meloche S. (2011). Extracellular signal-regulated kinases 1 and 2 regulate the balance between eccentric and concentric cardiac growth. Circ. Res..

[64] Purcell N.H., Wilkins B.J., York A., Saba-El-Leil M.K., Meloche S., Robbins J., Molkentin J.D. (2007). Genetic inhibition of cardiac ERK1/2 promotes stress-induced apoptosis and heart failure but has no effect on hypertrophy in vivo. Proc. Natl. Acad. Sci. USA.

[65] Tachibana H., Perrino C., Takaoka H., Davis R.J., Naga Prasad S.V., Rockman H.A. (2006). JNK1 is required to preserve cardiac function in the early response to pressure overload. Biochem. Biophys. Res. Commun..

[66] Gehani S.S., Agrawal-Singh S., Dietrich N., Christophersen N.S., Helin K., Hansen K. (2010). Polycomb group protein displacement and gene activation through MSK-dependent H3K27me3S28 phosphorylation. Mol. Cell..

[67] Gilsbach R., Preissl S., Gruning B.A., Schnick T., Burger L., Benes V., Wurch A., Bonisch U., Gunther S., Backofen R. (2014). Dynamic DNA methylation orchestrates cardiomyocyte development, maturation and disease. Nat. Commun..

[68] Kim J.Y., Kim K.B., Son H.J., Chae Y.C., Oh S.T., Kim D.W., Pak J.H., Seo S.B. (2012). H3K27 methylation and H3S28 phosphorylation-dependent transcriptional regulation by INHAT subunit SET/TAF-Ibeta. FEBS Lett..

[69] Awad S., Al-Haffar K.M., Marashly Q., Quijada P., Kunhi M., Al-Yacoub N., Wade F.S., Mohammed S.F., Al-Dayel F., Sutherland G. (2015). Control of histone H3 phosphorylation by CaMKIIdelta in response to haemodynamic cardiac stress. J. Pathol..

[70] Saadatmand A.R., Sramek V., Weber S., Finke D., Dewenter M., Sticht C., Gretz N., Wustemann T., Hagenmueller M., Kuenzel S.R. (2019). CaM kinase II regulates cardiac hemoglobin expression through histone phosphorylation upon sympathetic activation. Proc. Natl. Acad. Sci. USA.

[71] Joos J.P., Saadatmand A.R., Schnabel C., Viktorinova I., Brand T., Kramer M., Nattel S., Dobrev D., Tomancak P., Backs J. (2018). Ectopic expression of S28A-mutated Histone H3 modulates longevity, stress resistance and cardiac function in Drosophila. Sci. Rep..

[72] Awad S., Kunhi M., Little G.H., Bai Y., An W., Bers D., Kedes L., Poizat C. (2013). Nuclear CaMKII enhances histone H3 phosphorylation and remodels chromatin during cardiac hypertrophy. Nucleic Acids Res..

[73] Johannessen M., Delghandi M.P., Moens U. (2004). What turns CREB on?. Cell. Signal..

[74] Kasper L.H., Thomas M.C., Zambetti G.P., Brindle P.K. (2011). Double null cells reveal that CBP and p300 are dispensable for p53 targets p21 and Mdm2 but variably required for target genes of other signaling pathways. Cell Cycle.

[75] Watson P.A., Birdsey N., Huggins G.S., Svensson E., Heppe D., Knaub L. (2010). Cardiac-specific overexpression of dominant-negative CREB leads to increased mortality and mitochondrial dysfunction in female mice. Am. J. Physiol. Heart Circ. Physiol..

[76] Li J., Mahata B., Escobar M., Goell J., Wang K., Khemka P., Hilton I.B. (2021). Programmable human histone phosphorylation and gene activation using a CRISPR/Cas9-based chromatin kinase. Nat. Commun..

