# Supplementary material for: MSK-Mediated Phosphorylation of Histone H3 Ser28 Couples MAPK Signalling with Early Gene Induction and Cardiac Hypertrophy
Source: Cells. 2022 Feb 9;11(4):604. doi: 10.3390/cells11040604 (PMC8870627; doi:10.3390/cells11040604)
Supplement: Supplementary file 1 [file cells-11-00604-s001.zip › cells-1531389-supplementary.pdf]

Supplementary Figure S1

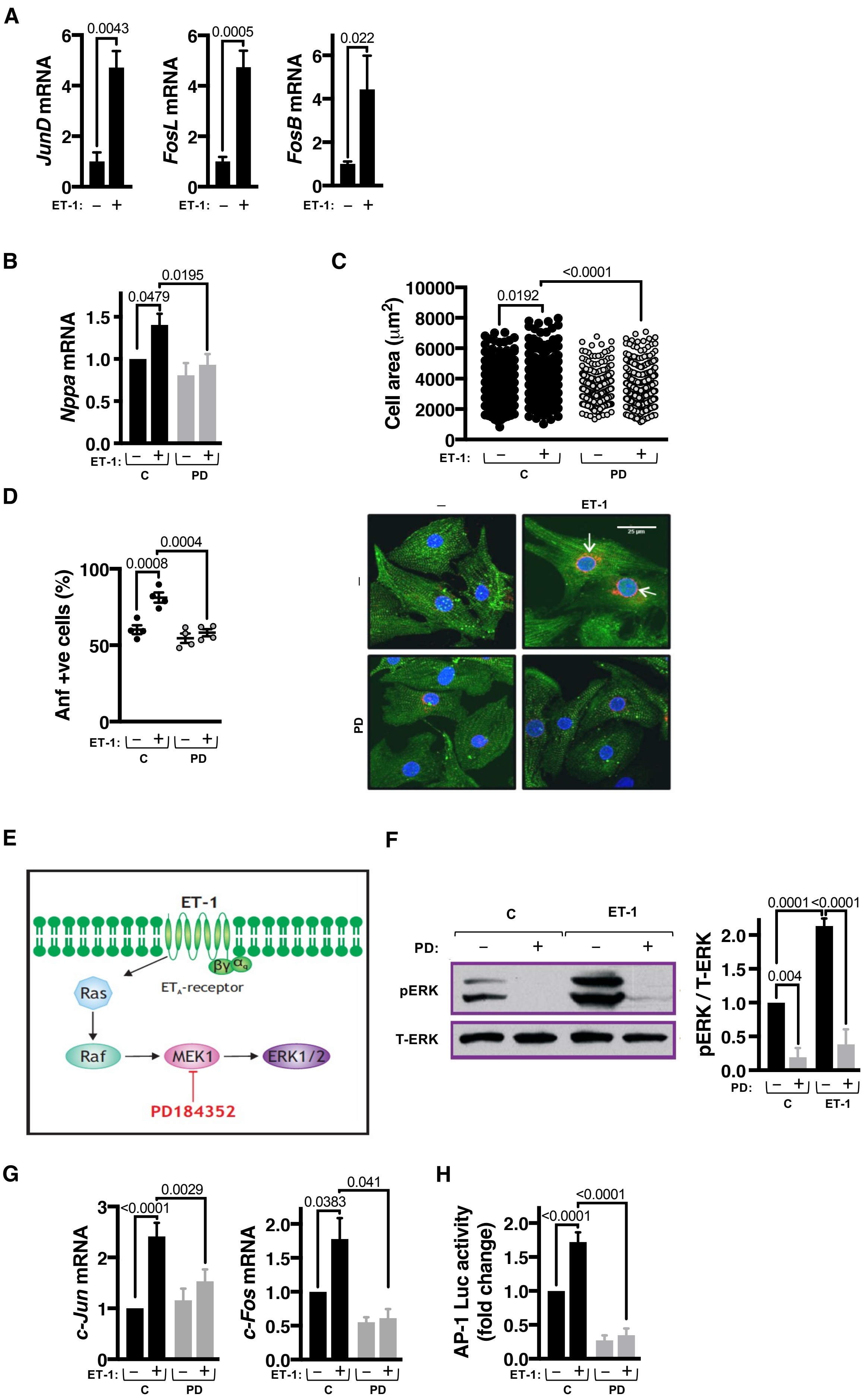

Supplementary Figure S1: IEG induction is activated by acute hypertrophic stimulation and attenuated by suppression of ERK signalling in vitro

A. RT-qPCR gene expression analysis of *JunD, FosL* and *FosB* mRNA in NRVMs +/- ET-1 treatment for 15 min. N=4. **B.** RT-qPCR gene expression analysis for *Nppa*/Anf mRNA in NRVMs +/- 30 min pre-treatment with PD184352 (PD). N=5 biological replicates (defined as 5 different NRVM preparations from different litters) **C.** Cell area (*μ*m2) as a measure of CM hypertrophy in NRVMs +/- PD +/- ET-1. N=4, 80-125 cells pers sample. **D.** Immunostaining for Anf (red), sarcomeric alpha-actinin (α-Act) (green) and nuclear stained with DAPI (blue) in NRVMs +/- PD +/- ET-1. Quantification (left) represents individual mean data points for N=4, representative images (right). The scale bar represents 25 µm. **E.** Schematic of the kinase signalling cascade induced by ET-1 stimulation resulting in ERK1/2 activation. Strategy used to inhibit ERK signalling via inhibition of MEK1 by PD184352 (PD) is indicated. **F.** Immunoblot analysis for phosphorylated ERK in NRVMs +/- PD +/- ET-1. Left: Representative immunoblot for phosphorylated ERK (pERK), normalised to total ERK (T-ERK). Right: Quantitation of relative pERK normalised to T-ERK. N=5. **G.** RT-qPCR analysis of *c-Jun* and *c-Fos* mRNA expression in NRVMs +/- PD +/- ET-1. For *c-Jun* (Left), N=4. For *c-Fos* (Right), N=3. **H.** AP-1 luciferase assay as a readout of AP-1 transcriptional activity in NRVMs +/- PD +/- ET-1 relative to untreated. For C and C ET-1, N=8. For PD and PD ET-1,N=3.

Supplementary Figure S2

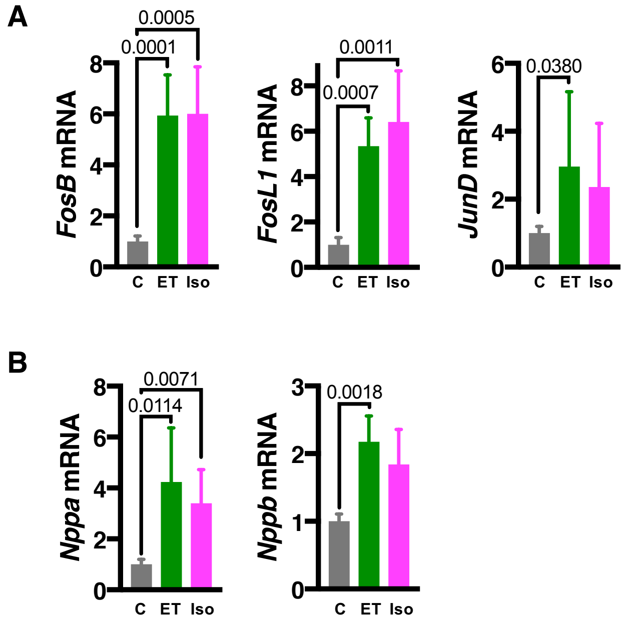

Supplementary Figure S2: Histone H3S28 phosphorylation is associated with induction of expression of IEG and hypertrophy-related genes.

A. RT-qPCR analysis of immediate early genes *JunD, FosL1, FosB* and of *Smarca4* (gene encoding BRG1) mRNA in hearts from adult male Wistar rats that were administered ET-1 or Iso through jugular vein administration and sacrificed 15 min later. C, N=7, ET-1, N=8 Iso, N=6. **B.** RT-qPCR expression analysis for cardiac hypertrophy-associated foetal genes *Nppa*/*Anf* and *Nppb*/*Bnp* mRNA in adult male Wistar rats that were administered ET-1 or Iso through jugular vein administration and sacrificed 15 min later. C, N=6; ET-1, N=6; Iso, N=7.

Supplementary Figure S3

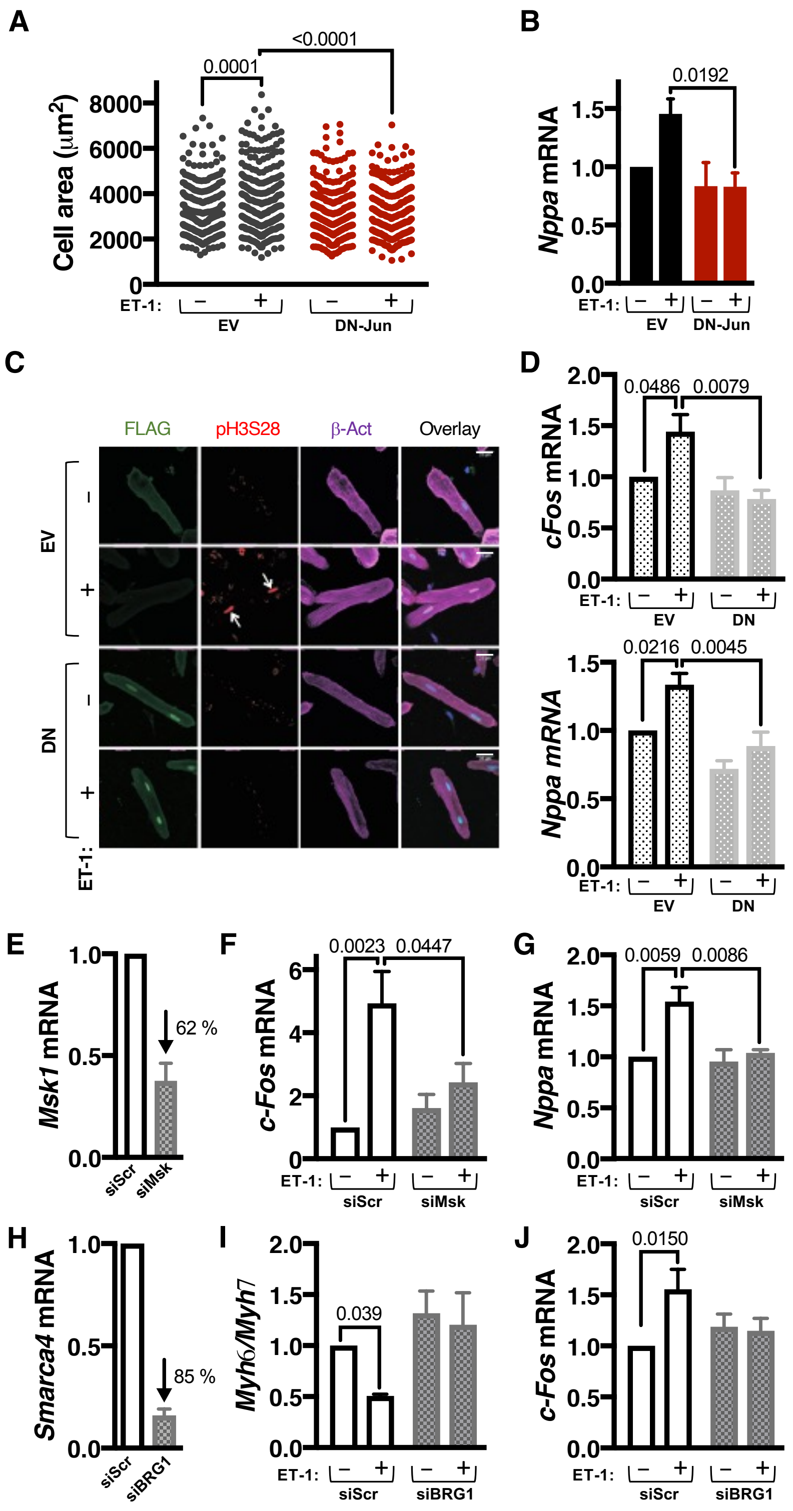

Supplementary Figure S3: IEG induction is a conserved feature of hypertrophy in adult CM

A. Cell area (μm^2) measurements for NRVMs infected with either control or DN-Jun (dominant negative, kinase dead) adenoviral vectors +/- ET-1. Individual data points are represented by N=4, 80-100 cells per sample. **B**. RT-qPCR analysis of *Nppa*/Anf mRNA expression in NRVMs +/- DN-Jun +/- ET-1, N=4. **C.** Representative confocal images of immunostained ARVMs infected with adenovirus expressing FLAG-tagged DN-MSK (kinase dead). ARVMs are stained for FLAG (green), pH3S28 (red) and β-Act (purple). FLAG-tagged DN-MSK is enriched in the nuclei in ARVMs. **D.** RT-qPCR analysis of *c-Fos* (Top) and *Nppa*/Anf (Bottom) mRNA in ARVMs infected with EV or DN-MSK1 +/- ET-1 treatment for 15 min. For *c-Fos* mRNA (top), EV ctrl and EV ET-1, N=4. For DN-MSK ctrl and DN-MSK ET-1, N=3. For *Nppa*/Anf mRNA (bottom), N=3. Scale bar = 25 μm. **E.** Analysis of siRNA-mediated knockdown of *Msk1* in NRVMs. RT-qPCR analysis of *Msk1* mRNA expression in NRVMs transfected with scr or siMsk siRNA is shown. N=3. **F.** RT-qPCR analysis of *c-Fos* mRNA in NRVMs transfected with scr or siMsk siRNA +/- ET-1 for 15 min. N=4. **G.** RT-qPCR analysis of *Nppa/Anf* mRNA expression in NRVMs transfected with scr or siMsk siRNA +/- ET-1 for 15 min. N=3. **H.** Analysis of *Smarca4* knockdown in NRVMs. *Smarca4/Brg1* mRNA abundance was measured by RT-qPCR in NRVMs transfected with siRNA targeting Smarca3 (siBrg1) and compared with NRVMs transfected with scrambled control (scr) siRNA. N=3. **I.** RT-qPCR analysis of *Myh6/Myh7* mRNA expression in NRVMs transfected with scr or siBrg1 siRNA +/- ET-1 for 15 min. N=5. **J.** *c-Fos* mRNA expression in NRVMs transfected with scr or siBrg1 siRNA +/- ET-1 for 15 min. N=6.

Supplementary Figure S4

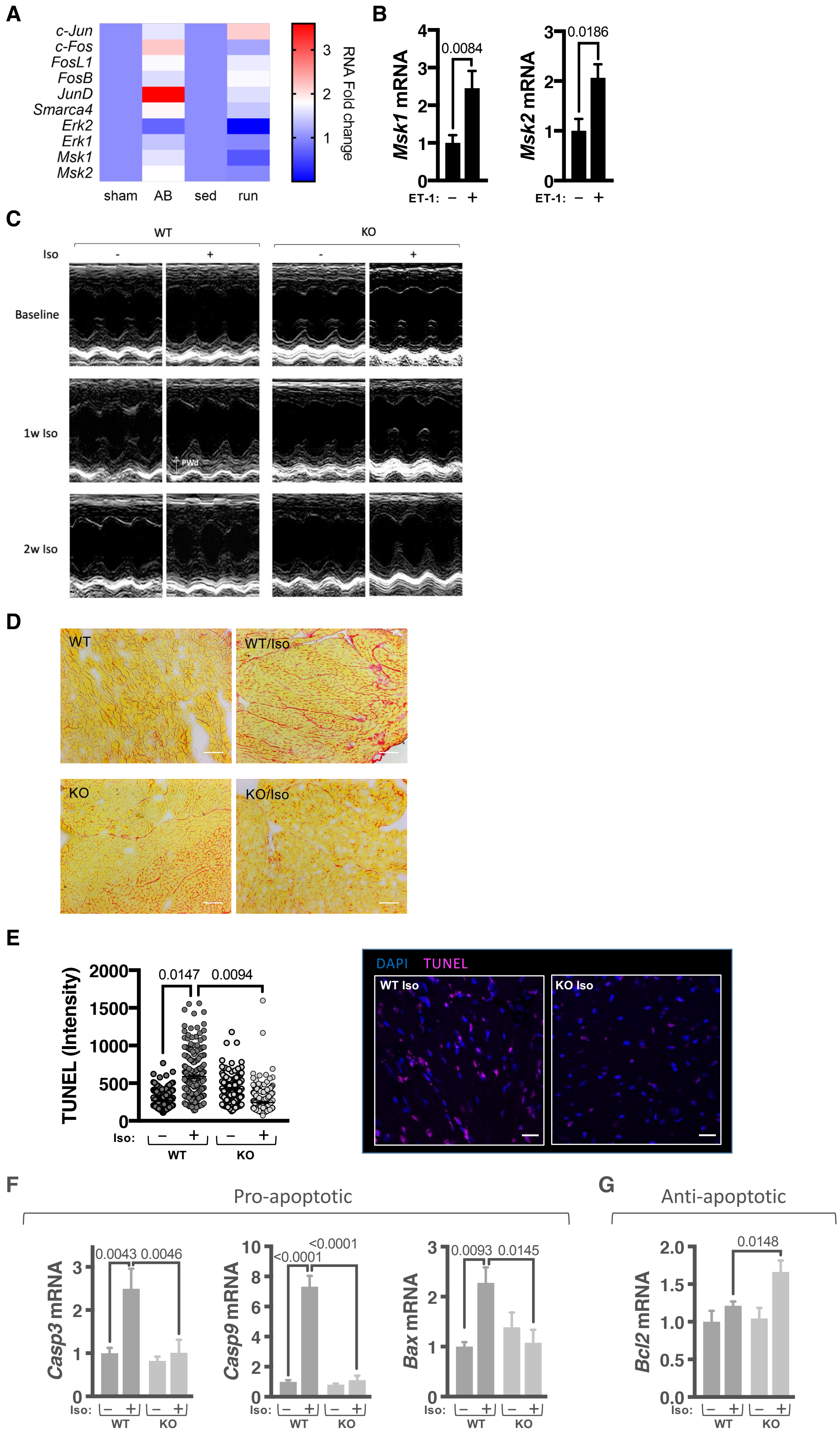

Supplementary Figure S4: Genetic MSK inhibition attenuates IEG activation and cardiomyocyte hypertrophy in vivo

A. Heat map showing expression of immediate early gene (*c-Jun, c-Fos, FosL1, FosB, JunD*), *Smarca4/Brg1, Erk1/2* and *Msk1/2* in left ventricular PCM-1+ve cardiomyocyte RNA-seq data in models of pathological (Ascending aortic banding, AB) or physiological (treadmill training, run) in male Sprague Dawley rats. Data extracted from [41]. Data available at GEO GSE66653. **B.** RT-qPCR analysis of *Msk1* and *Msk2* mRNA expression in NRVMs treated with ET-1 for 24 h. N=4. **C.** Representative M-mode echocardiogram from WT/*Msk1/2* KO mice +/- Iso for 1 week. Echocardiographic measurements indicated. PWd=posterior wall dimension end diastole. **D.** Representative left ventricular tissue sections from WT/*Msk1/2* KO mice +/- Iso for 2 week showing Picro sirius red staining for collagen. The scale bar indicates 50 μm. **E.** Terminal deoxynucleotidyl transferase dUTP nick end labelling (TUNEL) staining of left ventricular cardiac sections in *Msk1/2* KO mice and wild type littermates +/- Iso infusion for 2 week. TUNEL assay measures fragmented DNA as a mark of apoptosis. Left: Quantification of TUNEL in cardiac nuclei. Right: Representative immunostaining images for TUNEL (green) and nuclei are stained with DAPI (blue). N=3, 100-250 cells per sample. Scale bar indicates 20 μm. **F.** RT-qPCR analysis of pro-apoptotic marker genes caspase 3 (*Casp3*), caspase 9 (*Casp9*) mRNA expression in left ventricle from *Msk1/2* KO mice and wild type littermates +/- Iso infusion for 2 week. WT ctrl, N=5; WT Iso, N=4; KO ctrl, N=5; KO Iso, N=5. **G.** RT-qPCR analysis of the expression of the anti-apoptotic marker gene *Bcl2* mRNA expression in left ventricle from *Msk1/2* KO mice and wild type littermates +/- Iso infusion for 2 week. WT ctrl, N=5; WT Iso, N=4; KO ctrl, N=5; KO Iso, N=5.

Supplementary Tables

Table S1: Antibody information and dilutions used.

IF = immunofluorescence, IB = immunoblotting

Antibody	Order Information	Technique	Dilution Used
ANF	Bachem T-4014	IF	1:500
pERK	Cell signalling technology (CST), 9106S	IB	1:1000
T-ERK	BD Biosciences, 610031	IB	1:5000
α-Actinin	Merck, A7811	IB	1:2000
		IF	1:100
B-Actin	Abcam, 52219	IF	1:1000
Histone3	Merck, H0164	IB	1:10 000
Phos H3S10	CST, 9701S	IB	1:1000
Phos H3S28	Sigma Aldrich, H9908	IB	1:2000
		IF	1:1000
Phos H3S28	Merck, 07-145	ChIP	1:100
PCM1	Merck, Prestige, HPA023370	IF	1:500
Nesprin1	MANNES1A (7A12) kindly donated to use by Glenn Morris) (Randles et al., 2010)	IF	1:100
pMSK	CST, 9591S	IB	1:1000
cFos	Abcam, ab190289	IF	1:250
FLAG	Merck, FF3165	IB	1:2000
		IF	1:100
**Secondary Antibodies**			
HRP-conjugated antibodies	Jackson Laboratories	IB	1:10 000
Alexa Fluor 488	Thermo Fisher Scientific, R37116, A-11029	IF	1:500
Alexa Fluor 568	Thermo Fisher Scientific, A-11036	IF	1:500
Alexa Fluor 647	Thermo Fisher Scientific, A-21244	IF	1:500

Table S2: Primer sequence information

Primers were ordered from Merck or Integrated DNA Technologies as lyophilized custom oligos, purified by desalting.

Gene target	Species	Sequence
**RTqPCR**		
GAPDH	Rat	F-CAAGATGGTGAAGGTCGGTGT<br>R-GGTCGTTGATGGCAACAATG
	Mouse	F-AATGGTGAAGGTCGGTGTGAAC<br>R-TCGTTGATGGCAACAATCTCC
	Human	F-GAGTCAACGGATTTGGTCGT<br>R-GACAAGCTTCCCGTTCTCAG
TBP	Rat	F-TGACTCCTGGAATTCCCATC<br>R-TGTGTGGGTTGCTGAGATGT
	Mouse	F-GGGGAGCTGTGATGTGAAGT<br>R-CAGGAGAACATGGCAGACAA
	Human	F-TATAATCCCAAGCGGTTTGC<br>R-GCTGGAAAACCCAACTTCTG
YWHAZ	Rat	F-ACCCACTCCGGACACAGAAT<br>R- AGGCTGCCATGTCATCGTA
	Mouse	F-CAGAAGACGGAAGGTGCTGAGA<br>R-CTTTCTGGTTGCGAAGCATTGGG
	Human	F-CGAGATCCAGGGACAGAGTC<br>R-GGATGTTCTGTGTCCGGAGT
18S	Rat/Mouse	F-ATCCATTGGAGGGCAAGTC<br>R-CGCTCCCAAGATCCAACTAC
	Human	F-AAACCACAGGCAAACACCTC<br>R-GCACTTTGGGTGGTCAAGTT
RPL32	Rat/Mouse	F-GGCCAGATCCTGATGCCCAAC<br>R-CAGCTGTGCTGCTCTTTCTAC
	Human	F-AGGCATTGACAACAGGGTTC<br>R-GACGTTGTGGACCAGGAACT
SDHA	Rat	F-TCGCACTGTGCATAGAGGAC<br>R-ATGCCTGTAGGGTGGAACTG
	Mouse	F-AAGGCAAATGCTGGAGAAGA<br>R-TGGTTCTGCATCGACTTCTG
	Human	F-TCGCACTGTGCATAGAGGAC<br>R-GCCTGTAGGGTGGAACTGAA
LMNA	Rat	F-TGAGTACAACCTGCGCTCAC<br>R-TGTGACACTGGAGGCAGAAG
	Mouse	F-GCACCGCTCTCATCAACTCC<br>R-TCTTCTCCATCCTCGTCGTCA
	Human	F-CTACACCAGCCAACCCAGAT<br>R-GGTCGAAGGACAGAGACTGC
Tuba 6	Mouse	F-GGCAGTGTTCGTAGACCTGGA<br>R-TTATTGGCAGCATCCTCCTTG

cFOS	Rat	F-CCGACTCCTTCTCCAGCATG<br>R-GTGGAGATGGCTGTCACCGT
	Mouse	F-GGGAATGGTGAAGACCGTGTCA<br>R-GCAGCCATCTTATTCCGTTCCC
	Human	F-GCCTCTCTTACTACCACTCACC<br>R-AGATGGCAGTGACCGTGGGAAT
cJUN	Rat	F-TTGAAAGCGCAAAACTCCGA<br>R-GTTAGCATGAGTTGGCACCC
	Mouse	F-CAGTCCAGCAATGGGCACATCA<br>R-GGAAGCGTGTTCTGGCTATGCA
	Human	F-CCTTGAAAGCTCAGAACTCGGAG<br>R-TGCTGCGTTAGCATGAGTTGGC
JUND	Rat	F-GACATGGACACGCAGGAAC<br>R-TCTGGCTTTTGAGGGTCTTG
	Human	F-ATCGACATGGACACGCAGGAGC<br>R-CTCCGTGTTCTGACTCTTGAGG
FOSB	Rat	F-AAACAAACAAAACCGCAAGG<br>R-GGCGGTCAGACAGAAGAGTC
	Human	F-TCTGTCTTCGGTGGACTCCTTC<br>R-GTTGCACAAGCCACTGGAGGTC
FOSL	Rat	F-AGAGCTGCAGAAGCAGAAGG<br>R-GCTGGTACCACCTGTGTCCT
	Human	F-GGAGGAAGGAACTGACCGACTT<br>R-CTCTAGGCGCTCCTTCTGCTTC
SMARCA4	Rat	F-CTACAGGAGCGGGAGTACAG<br>R-TGGTTGCTTTGGTTCGAAGG
	Mouse	F-GAAAGTGGCTCTGAAGAGGAGG<br>R-TCCACCTCAGAGACATCATCGC
	Human	F-CAAAGACAAGCACATCCTCGCC<br>R-GCCACATAGTGCGTGTTGAGCA
MSK1	Rat	F-TCTATGTTGGAGAGATCGTGCTTG<br>R-AATCTGTCAGCACCACATGGC
	Mouse	F-TGGTCCATAGCACCTCTCAGCT<br>R-CTCTCCGCCATTCAGAAGTTCC
	Human	F-CCTGGAACACATTAGGCAGTCG<br>R-CACCTCATGCTCTGTGAAACGC
MSK2	Rat	F-ATCCTGGACTATGTGAGCGG<br>R-CTAGGGCCAGCACAATCTCT
	Mouse	F-CAACGTGGTGAATCTGCATGAGG<br>R-AGCCGCTTCTTGCGGATGTGTT
	Human	F-TGTGGGCAACTTTGCGGAGGAA<br>R-GAGAATGGAGGGTGCCACAAAG
MYH6	Rat	F-ACAGAGTGCTTCGTGCCTGAT<br>R-CGAATTTCGGAGGGTTCTGC
	Mouse	F-CAGAGGAGAAGGCTGGTGTC<br>R-CTGCCCCTTGGTGACATACT
	Human	F-CTGGGCAAGTCCAACAATTT<br>R-CCAGCCCAGGATGTTGTAGT
MYH7	Rat	F-GCCTACAAGCGCCAGGCT

		R-CATCCTTAGGGTTGGGTAGCA
	Mouse	F-CTTCAACCACCACATGTTCG<br>R-TCTCGATGAGGTCAATGCAG
	Human	F-GTGAAAGTGGGCAATGAGT<br>R-TGGTGAAGTTGATGCAGAGC
Nppa/Anf	Rat	F-CGTATACAGTGCGGTGTCCAAC<br>R-CCTCATCTTCTACCGGCATC
	Mouse	F-GTGCGGTGTCCAACACAGAT<br>R-TTCCTCAGTCTGCTCACTCAGG
Nppb/Bnp	Rat	F-GGTCTCAAGACAGCGCCTTCC<br>R-CTTCCTAAAACAACCTCAGCCCGTC
	Mouse	F-GCCAGTCTCCAGAGCAATTC<br>R-GTTCTTTTGTGAGGCCTTGG
COL1A1	Mouse	F-GCCAAGAAGACATCCCTGAA<br>R-GCCATTGTGGCAGATACAGA
Casp3	Mouse	F-GGGCCTGTTGAACTGAAAAA<br>R-CCGTCCTTTGAATTTCTCCA
Casp9	Mouse	F-GCCAGAGGTTCTCAGACCAG<br>R-AAGCCGTGACCATTTTCTTG
Bax	Mouse	F-TGCAGAGGATGATTGCTGAC<br>R-GATCAGCTCGGGCACTTTAG
Bcl-2	Mouse	F-GGACTTGAAGTGCCATTGGT<br>R-AGCCCCTCTGTGACAGCTTA
**ChIP-qPCR**		
cFos promoter	Rat	F-CCTTGCGCTGCACCCTCAGA<br>R-CGGCCGTGGAAACCTGCTGA
cJun promoter	Rat	F-TGTAGGAGCGCAGCGGAGCA<br>R-CCCACCCGTCGCCATGGAGA
cFOS promoter	Human	F-TGTTATAAAAGCAGTGGCTGCG<br>R-TCTTGGCTTCTCAGATGCTCG
cJUN promoter	Human	F-TCTCTCCGTCGCAACTTGTC<br>R-ACGCAGCAGTTGCAAACATT
SMARCA4 promoter	Human	F-GGGAAGTTTTGCAGAGAAGGC<br>R-CCTGGGAACCGCTTTGATCC

Table S3: Human non-failing donor samples for cardiomyocyte isolation

Subject	
Age	70.5±4.6
Sex	4 Female
BMI	25±2
Medications	Antibiotics, corticosteroids, Vasopressor, insulin.

Samples used in Table S3 were used to generate data in Figures 4A-B.

Table S4: Human control and hypertrophic left ventricular cardiomyocyte nuclei

	Control	Hypertrophic
Age	48.8±5.8	51±5.96
Heart Weight (g)	421±46	589±89
BMI	24.6±2.30	27.8±2.68
Cause of death	Suicide (2)<br>Intoxication (2)<br>Fall (1)	Natural causes (3)

Samples in Table S4 were used to generate data in Figure 4C-G.

Materials and Methods

Reagents

Chemicals were from Merck (Darmstadt, Germany) and molecular biology reagents were from (Thermo Fisher Scientific, MA, USA), unless stated otherwise. Tables of antibodies and primers used in this study are included in Supplementary Tables S1and S2, respectively.

Animal experiments

All experiments involving animals were in accordance with the European Directive 2010/63/EU. Experiments were performed in accordance with the UK Home Office and institutional guidelines or were approved by the Ethical Committee for Animal Experiments of the KU Leuven (Belgium). The *Msk1/2* null animals have been previously described [1,2]. Hypertrophic remodelling was induced in 8-10 week old male mice animals by administration of isoproterenol (Iso, Merck) at 10 mg/kg/day for one week via osmotic mini-pumps (Alzet) implantation as previously described and under project license P3A97F3D1 [3]. Jugular vein infusion of ET-1 and Iso was performed as previously described [4] using an approved experimental protocol (license number P055/2017) approved by the Ethical Committee for Animal Experiments of the KU Leuven (Belgium), explained in detail later. Male Sprague Dawley rats subjected to six weeks of ascending aortic banding or a six-week treadmill training program were previously used to generate cardiomyocyte-specific nuclear (PCM-1 positive) RNA-sequencing data as previously described [5]. RNA sequencing data was re-purposed for this study, focusing on the panel of IEGs. The sequencing data are available in the NCBI's Gene Expression Omnibus (GEO) database (GEO GSE66653).

Echocardiography

Mice were anaesthetised with Avertin (200 mg/kg). Cardiac function was assessed by transthoracic 2D M-mode echocardiography using an Acuson Sequoia C256 ultrasound system (Siemens, Munich, Germany) as previously described [3].

Preparation of neonatal rat ventricular cardiomyocytes (NRVMs)

Primary neonatal rat ventricular cardiomyocytes (NRVMs) were isolated from 3-4 day old male and female Wistar pups and cultured as described previously [6]. Cultures were > 95 % pure. NRVMs were seeded at a density at which they exhibited spontaneous and synchronous beating throughout the experiment. 48 h after seeding, NRVMs were washed into serum-free medium (DMEM/M199 4:1, 1 mM sodium pyruvate, 5.5 µg/mL transferrin, 5 ng/mL sodium selenite, 1 X Antibiotic-Antimycotic (Thermo Fisher Scientific), and 3 µM cytosine β-D-arabinofuranoside (araC) and serum-starved for 24 h. NRVMs were subsequently stimulated with the agents described. Adenoviral infections were performed by incubation with a volume of virus-containing serum-free medium sufficient to cover the cells for 4 h. Agonist treatments diluted in serum-free medium were applied 24 h post-infection with adenovirus. Endothelin-1 (ET-1; Merck), isoproterenol hydrochloride (Iso; Merck) and PD184352 (PD; Merck) treatments were performed at a final concentration of 100 nM, 10 nM and 1 µM respectively. All cellular treatments with PD were pre-treated with PD for 30 min prior to hypertrophic agonist application (ET-1/Iso). Control cellular experiments (no treatment) were treated with the same volume of vehicle only (DMSO for ET-1 and PD).

Isolation and culture of adult rat ventricular cardiomyocytes (ARVMs)

Male Wistar rats (Harlan; ~200 g) were anaesthetised by CO_2 inhalation and sacrificed by cervical dislocation. ARVMs were isolated by collagenase digestion following Langendorff perfusion as previously described and cultured in adult cardiomyocyte medium (M199, 1 % penicillin-streptomycin-l-glutamine, 0.2 % bovine serum albumin (BSA)) on laminin-coated (25 µg/ml) dishes [7]. Adenoviral infections were performed for 12 h in a minimal volume of virus-containing medium. For experiments involving acute stimulation with ET-1 and Iso, cells in Tyrode's solution ((in mmol/L: NaCl 137, KCl 5.4, $MgCl_2$ 0.5, $CaCl_2$ 1.8, NaHEPES 11.8, and glucose 10; pH 7.4) were plated onto laminin-coated 8 well Nunc cover glasses cells, allowed to attach for 1 h at 37 °C, after which the Tyrode's

solution was replaced with Tyrode's solution containing DMSO vehicle or Tyrode's solution containing 10 nM PD. After 20 min, buffer was exchanged for Tyrode's solution containing 100 nM ET-1 or 10 nM Iso ± PD. After 15 min, dishes were placed on ice and processed for immunostaining and imaging.

Isolation of human ventricular cardiomyocytes

Cardiomyocytes were isolated as previously described from non-failing heart tissue samples obtained from donor hearts not suitable for transplantation [8]. Donor human tissue was collected under a study protocol approved by the ethical committee of UZ Leuven (S58824), which conformed to the Helsinki declaration and was conducted in accordance with the prevailing national and European Union regulations on the use of human tissues. Donor information is displayed in Supplementary Table S3. The explanted hearts were collected in ice-cold modified Tyrode's solution at the time of surgery (in mM: NaCl 130, KCl 27, HEPES 6, $MgSO_4$ 1.2, KH_2PO_4 1.2, glucose 50; pH 7.2 with NaOH) and transported to the lab. A wedge of the left ventricle with its perfusing coronary artery was cannulated. If possible, a wedge from the left anterior descending artery was cannulated, otherwise a left circumflex branch was used. The artery and the tissue were perfused at 37 °C with a Ca^{2+}-free Tyrode's solution (in mM: NaCl 130, KCl 5.4, HEPES 6, $MgSO_4$ 1.2, KH_2PO_4 1.2, glucose 20; pH 7.2 with NaOH) for 30 min followed by enzyme perfusion for 40 min (collagenase A, Roche and protease XIV, Merck in Ca^{2+}-free solution) and after digestion, perfused with low Ca^{2+} Tyrode's (Ca^{2+}-free solution with 0.18 mM $CaCl_2$ added). The digested tissue was minced, the suspension was filtered and the isolated cardiomyocytes were resuspended in normal Tyrode's solution. After isolation, the cells were allowed to recover for 1 h before starting experiments or fixation. For experiments involving stimulation with Iso or ET-1, cells were plated onto poly-L-lysine coated 8 well Nunc cover glasses and allowed to attach for 1 h at 37 °C. After this period the Tyrode's solution was replaced with Tyrode's containing DMSO vehicle or Tyrode's solution containing 1 μM PD. After 20 min, buffer was exchanged for Tyrode's solution containing 100 nM ET-1 or 10 nM Iso ± PD. After 15 min, dishes were placed on ice and processed for immunostaining and imaging.

Isolation of human cardiomyocyte nuclei

Nuclei from post-mortem left ventricular tissue were isolated and flow sorted according to pericentriolar material 1 (PCM-1) staining as previously described [5]. 500,000 nuclei were sorted into 1 ml TRIzol reagent for RNase inhibition prior to RNA isolation. Human LV samples were obtained from the KI Donatum, Karolinska Institutet, Stockholm, Sweden, with permission for the analysis of human tissue for research purposes granted by the Regional Ethics Committee in Stockholm, Sweden. Donor information from which LV CM nuclei were isolated is displayed in Supplementary Table S4.

High content analysis of NRVM hypertrophy

Analysis of surface area of NRVM was carried out as previously described [6]. Briefly, NRVMs for immunofluorescence were cultured and fixed in black 96-well imaging microplates (BD Biosciences, , NJ, USA). NRVMs were immunostained with primary antibodies against α-Act and ANF and detected using Alexa Fluor 488 and 568-coupled secondary antibodies (Supplementary Table S1). After immunostaining, nuclei were labelled with Hoescht (1 μg/ml in PBS for 20 min). Images were captured using a BD Pathway 855 high-content imaging system and Attovision software. Cell planimetry was performed using ImageJ by drawing around the edge of the cells (NIH). At least 400 cells from three independent experiments were analysed. These images were also used for quantification of ANF protein expression as determined by counting the number of NRVM exhibiting a peri-nuclear ring of ANF.

Confocal imaging of immunostained NRVM and ARVM

NRVMs were cultured in 16-well chamber slides (Nunc) and immunostained as for high content imaging using antibodies described in Supplementary Table S1. Slides were mounted onto coverslips using VECTASHIELD Mounting Medium containing DAPI and sealed with clear nail varnish.

For immunostaining of ARVMs and isolated human CMs, cells were fixed and permeabilised in ice-cold 100 % methanol and incubated at -20 °C for 10 min. Methanol was washed from the coverslips

twice with PBS and further permeabilisation performed by the addition of 0.5 ml ice-cold 100 % acetone and incubation at -20 °C for 1 min. Following an additional two washes in PBS, antibody labelling was performed as described for NRVM. To further reduce background staining, blocking and secondary antibody buffers contained 1 % BSA in addition to goat serum. Images were captured using a Nikon A1R confocal microscope equipped with a Plan Fluor DIC H N 40x/1.3 NA oil immersion objective (human CM and ARVM treated with hypertrophic agonists) or using an Olympus FV1000 point scanning microscope attached to an Olympus IX81, equipped with a 40x/1.3 NA UPlanFI oil immersion objective (ARVM expressing MSK constructs).

Confocal imaging of immunostained cardiac tissue sections

Immunofluorescence analysis of cardiac sections was performed as previously described [5]. Fresh or previously snap frozen were embedded in Tissue-Tek optimum cutting temperature (OCT) in Tissue-Tek® Cryomold® Molds (15x15x15 mm) and flash-frozen in liquid nitrogen-cooled isopentane (VWR). 10 µm sections were cut using a Leica cryostat and attached to SuperFrost Plus™ slides (VWR). Slides were frozen at -20 °C prior to immunostaining. Sections were thawed and rehydrated in phosphate buffered saline (PBS) for 5 min, followed by 15 min fixation in 4 % paraformaldehyde (PFA). After three washes (5 min each) in PBS, sections were permeabilised for 30 min in 0.2 % Triton X-100 in PBS (PBS-TX), then washed twice with PBS. Non-specific protein binding sites were blocked by incubation in PBS-TX containing 3 % BSA, 5 % goat serum or 5 % Chemibloc for 1 h. Sections were subsequently incubated overnight at 4°C in blocking buffer with primary antibodies as per Supplementary Table S1. Samples were washed extensively in PBS-TX, and incubated with Alexa Fluor® secondary antibodies (Invitrogen) at 1:500 in PBS-TX for 1 h at room temperature. For detection of apoptosis in tissue sections, the TACS® 2 TdT-DAB In Situ Apoptosis Detection Kit was used (Bio-Techne Ltd., Abingdon, UK). Prior to imaging, sections were mounted in VECTASHIELD with DAPI (Vector Labs, CA, USA). Images were captured using a Nikon A1R confocal microscope equipped with a Plan Fluor DIC H N 40x/1.3 NA oil immersion objective. Image stacks were collected over a 2 µm stack thickness (0.2 µm z-step). Image stacks were analysed with Volocity Image analysis software (version 6.2.1, Perkin Elmer, MA, USA). Intensity of labelling of targets of interest was quantitated specifically in cardiac myocyte nuclei, which were identified as staining positive for DAPI and for either PCM-1- or Nesprin, as previously described [41].

Picro Sirius Red staining for fibrosis analysis

10 µm thick sections were cut from OCT embedded tissue as above. Subsequently, sections were rehydrated and stained for collagen using a Picro Sirius red staining kit (PolySciences, Hirschberg an der Bergstraße, Germany). After staining, sections were mounted in dibutylphthalate polystyrene xylene mounting medium. Images were acquired using a Zeiss Axioplan microscope configured with an Axiocam HrC camera. Polarization microscopy was performed on the Sirius red stained sections to visualize collagen type I and III based on the birefringence properties of collagen. The degree of fibrosis was quantified using Zeiss Axiovision analysis software (Version 4.6).

Histone isolation by acid extraction

NRVMs in 6-well dishes were washed once in ice-cold PBS. 0.5 ml of fresh ice-cold PBS was added to each well, the cells scraped and placed in pre-chilled 1.5 ml tubes. Cells were pelleted by centrifugation (10 min, 300 xg, 4 °C). PBS was removed and the pellet re-suspended in 1 ml hypotonic lysis buffer. The resuspended cells were incubated on a rotator at 4 °C, 30 rpm for 30 min. At the end of this incubation, intact nuclei were pelleted by centrifugation (10 min, 10000 xg, 4 °C). Nuclei were then resuspended in 400 µl 0.2 M H_2SO_4 by pipetting and vortexing. Histones were acid extracted overnight at 4 °C on a rotator at 30 rpm. Following acid extraction, nuclear debris was removed by centrifugation (10 min, 16000 xg, 4 °C) and the supernatant containing isolated histones transferred to a pre-chilled tube. To precipitate proteins, 100 % trichloroacetic acid was added to the supernatant in a drop-wise manner to achieve a final concentration of 25 %. The tube was gently inverted and then incubated on ice for 6 h. At the end of this period, precipitated proteins were recovered by centrifugation (10 min, 16000 xg, 4 °C). The supernatant was aspirated and acid removed from the tube by washing the pellet in 300 µl ice-

cold acetone. After centrifugation (5 min, 16000 xg, 4 °C) and removal of the supernatant, the acetone wash and spin were repeated. Finally, the supernatant was gently removed and the pellet air-dried for 20 min at room temperature. The dried pellet was resuspended in 50 µl water and incubated overnight at 4 °C on a rotator at 30 rpm to maximise protein solubilisation.

Immunoblot analysis

Immunoblotting was performed as previously with minor modifications [5]. Cultures of NRVM were washed once in ice-cold PBS after which 80 µl pre-chilled RIPA buffer was added to the dish and incubated for 5 min on ice (25 mM Tris-HCl, pH 7.6, 150 mM NaCl, 0.1 % SDS, 1 % NP-40, 1 % Sodium Deoxycholate supplemented with 1 X Protease and Phosphatase inhibitor cocktails (Merck). The cell lysate was transferred to a pre-chilled tube and debris removed by centrifugation (5 min, 10 000 g, 4 °C). The supernatant was transferred to a clean tube and total protein concentration determined using the BCA assay (Thermo Fisher Scientific). Equivalent amounts of protein (10-30 µg) were loaded and samples prepared with LDS sample buffer (Invitrogen) containing 2.5 % β-mercaptoethanol and boiled at 95 °C for 5 min. Proteins were resolved on pre-cast 4-12 % NuPAGE 1.5 mm 10 well SDS gels using MOPS running buffer (Thermo Fisher Scientific). Novex pre-stained sharp protein markers were used (Invitrogen). For detection of ERK or MSK, proteins were transferred to a PVDF (0.45 µm, Merck) membrane. For detection of histone H3 and its modified forms, proteins were transferred to nitrocellulose (0.2 µm, Whatman/Cytiva, MA, USA). Proteins were detected with appropriate primary antibodies and HRP-conjugated secondary antibodies (Supplementary Table S1). Immunoreactive bands were detected by enhanced chemiluminescence (Thermo Fisher Scientific).

Reverse transcription quantitative PCR (RT-qPCR)

RNA was isolated from NRVMs and ARVMs using the RNeasy Micro Kit (Qiagen) and DNA removed by an on-column DNA digestion step. RNA was isolated from adult rat LV tissue, *Msk1/2* knockout (KO) mouse LV tissue and human CM using TRIzol reagent (Thermo Fisher Scientific).
500-750 ng RNA was reverse transcribed using Superscript II (Invitrogen), the final cDNA synthesis reaction diluted 1/10 - 1/20 in nuclease free water and stored at -20 °C until required. Primer sequences were as previously described, unless otherwise indicated (Supplementary Table S2), and were designed to span intron-exon boundaries to avoid amplification of genomic DNA [6]. The stability of a panel of reference genes was assessed using the GeNorm method for the experiments performed, and the most stable selected [9]. Three or four reference genes were selected for each set of experimental conditions for normalisation of gene expression qPCR analysis based on their stability for each set of samples and reaction conditions. Final primer concentration was 200 nM for all targets. Reactions were performed in triplicate. Reactions were performed on a LightCycler® 480 System (Roche, Basel, Switzerland) or on a CFX384 (Bio-Rad, CA, USA) in a 384-well format using Platinum SYBR Green qPCR SuperMix-UDG (Life Technologies). Expression analysis was carried out using the comparative ΔCt method as described [10].

Chromatin-immunoprecipitation (ChIP)

To NRVMs in 6-well dishes in culture medium, formaldehyde was added to a concentration of 1 % and incubated for 10 min on a rocking platform at room temperature. Cross-linking was terminated by the addition of 125 mM glycine for 10 min at room temperature. After washing once in ice-cold PBS, cells were collected into 0.5 ml PBS by scraping and subsequent centrifugation (5 min, 600 xg, 4 °C). Cell pellets were re-suspended in 1 ml ice-cold hypotonic membrane lysis buffer (50 mM Tris-HCl, pH 7.5, 5 mM EDTA, 140 mM NaCl, 1 % Triton X-100, 0.5 % NP-40 supplemented with 1 X Protease and Phosphatase inhibitor cocktails). The released nuclei were pelleted by centrifugation (3 min, 12000 xg, 4 °C) and then re-suspended in 200 µl SDS lysis buffer (50 mM Tris-HCl, pH 7.5, 10 mM EDTA, 1 % SDS supplemented with 1 X Protease and Phosphatase inhibitor cocktails). Cross-linked chromatin was fragmented by sonication using a pre-chilled Diagenode Bioruptor (NJ, USA) on the high power setting for three x 5 min cycles of 30 s 'on', 30 s 'off'. The sonication protocol produced fragments predominantly below 500 bp. Wash buffer + (10 mM Tris-HCl, pH 7.5, 140 mM NaCl, 1 mM EDTA, 0.5 mM EGTA, 1 % Triton X-100, 0.1 % SDS and 0.1% Na deoxycholate supplemented with 1 X Protease

and Phosphatase inhibitor cocktails) was then added to the lysate. After removal of remaining debris by centrifugation (10 min, 12000 xg, 4 °C), the supernatant (sonicated chromatin) was processed for immunoprecipitation.
Proteins of interest were precipitated using antibodies pre-conjugated to Dynabeads Protein A (Thermo Fisher Scientific). Antibodies used for ChIP are indicated in Supplementary Table S1. To this end, beads were washed in 4 changes of wash buffer and collected using a DynaMag Magnet. Per ChIP, 10 µl washed beads and 5 µg of antibody against BRG1 or phosphorylated histone H3S28 were added to 90 µl wash buffer and incubated for two h at 40 rpm on a rotator at 4 °C. For the negative control ChIP, beads were used in the absence of specific antibody.
Prior to IP, wash buffer was removed from pre-prepared antibody-bead complexes and 200 µl chromatin added to each tube. 200 µl chromatin was reserved from each experimental condition as an Input sample. Chromatin was incubated with the antibody-bead complexes overnight at 4 °C on a 40 rpm rotator after which unbound chromatin was washed from the beads. Wash buffer was used for the first two washes, followed by one wash in high-salt wash buffer (wash buffer + with 500 mM NaCl) and finally two washes in TE buffer. At the end of the washes, the solution was transferred to a fresh tube and TE buffer removed from the beads. Elution of chromatin from the beads and protein digest with proteinase K were combined into one step. To this end, 150 µl complete elution buffer (20 mM Tris-HCl, pH 7.5, 50 mM NaCl, 5 mM EDTA, 1% SDS and 100 µg/ml proteinase K (Sigma)) was added and the beads incubated for 4 h at 68 °C with shaking at 1300 rpm. The supernatant was removed from the tube and 150 µl elution buffer (complete elution buffer without SDS and Proteinase K) added. After a further 5 min incubation at 68 °C, the two supernatants were combined. Input samples were processed in parallel with the ChIP samples. DNA was purified from each supernatant using the QIAEX II Gel extraction kit (Qiagen) following the manufacturer's protocol. DNA was eluted in 40 µl buffer EB and stored at -20 °C.
Precipitated DNA for each experimental condition and antibody was quantified by qPCR using SYBR-GreenER in 12.5 µl reactions performed in triplicate (Thermo Fisher Scientific). Sequences of primers used are given in Supplementary Table S2. Primer binding sites were selected that encompassed predicted transcription factor binding sites. qPCR was performed using a CFX96 (Bio-Rad) or Roche Light Cycler480 real-time PCR instrument and cycling parameters were taken from the manufacturer's instructions for SYBR-GreenER (Thermo Fisher Scientific).
The Ct values from triplicate technical replicates (n=3) from each sample were averaged to generate SampleCT and InputCt values. The values were analysed by expressing enrichment of the immunoprecipitated DNA for each antibody as a percentage of the input sample for the relevant experimental condition. ChIPs were repeated on at least 3 independent experimental samples.

Jugular vein infusion of endothelin-1/isoproterenol in Wistar rat

Experimental protocols were approved by the local ethical committee (Ethische Commissie, Dierproeven, KU Leuven), under license number P055/2017 and were performed as previously described [4]. 250-300 g Wistar (RccHan:WIST) male rats were obtained from Harlan (NL). Anesthesia was induced using ketamine and xylazine in combination (100 mg/kg ketamine, 10 mg/kg xylazine) by IP. Body temperature was maintained throughout the procedure with a heated mat (Sanitas).
A small area of chest was shaved with depilatory cream (Veet) and limbs secured with tape. A small incision was made just above and to the right hand side of the sternum and the skin stretched thin with a hemostat to make the jugular vein visible. A 30 gauge needle attached to a cannula (2F x 30 cm, green, Portex) was inserted into the jugular vein, just before it branches and the vein disappears under the pectoral muscle.
The cannula was attached to a 5 ml syringe and a dispensing pump (Harvard Apparatus) dispensing the required volume (300-500 µl) over a 15-min period. A slow steady release of the dosage in this manner was required to reduce the acute vasoconstrictive effect of a single rapid injection of the same dosage.
Endothelin-1 (Merck) was administered at a final dosage of 1000 ng/kg and isoproterenol hydrochloride (Merck) at 50 µg/kg. Final working concentrations prepared in sterile saline and vehicle-only controls (Ctrl) were administered the same volume of sterile saline over a 15 min period. On withdrawal of the needle, medical gauze was placed over the wound and pressure applied until bleeding stopped. The

wound was cleaned with iodine solution. Rats were sacrificed by cervical dislocation and the heart immediately removed for dissection. Whole hearts were removed and placed in ice cold PBS briefly to remove excess blood, dissected using a sterile surgical scalpel in PBS on ice and weighed on a microbalance before snap-freezing in liquid nitrogen and stored at -80 °C.

Adenoviral methods

Adenoviruses were produced and amplified in HEK293 cells and purified as previously described [6]. Adenoviruses to express the WT and catalytically dead D565A mutant (DN) of MSK1 were generated using the AdEasy method by sub-cloning the cDNA for MSK1 or its mutant from a pCMV5 backbone (kindly provided by Prof D Alessi, University of Dundee) into pShuttle CMV [11]. PacI digested recombinant plasmids were transfected into HEK293 cells and crude adenovirus harvested after 10-14 days. Adenoviruses for dominant negative (DN)-Jun and AP-1 luciferase were purchased from Vector Biolabs (PA, USA). All viruses were amplified in HEK293 cells, purified using the Vivapure Adenopack 100 (Sartorius, Gottingen, Germany) and titrated by end-point dilution in HEK293 cells.

Analysis of luciferase reporter activity

The AP-1 luciferase reporter was expressed using an adenoviral vector and luciferase activity determined using a luciferase assay kit from Promega (MA, USA) as previously described [42]. Briefly, cultures of NRVM in 48 well plates were infected in duplicate and agonist treatments applied for 24 h. After removal of medium, cells were lysed in 150 µl 1X cell culture lysis buffer (Promega). Luciferase activity present in 10 µl of lysate clarified by centrifugation was then quantitated in white 96-well luminometer plate (Microlumat Plus 1b 96V instrument, Berthold Technologies) using 50 µl of luciferase assay reagent (Promega).

Small interfering RNA (siRNA) knockdown

Stealth™ siRNAs were purchased from Invitrogen. To achieve sufficient knockdown of *Msk1* or *Brg1*, two siRNAs targeting different regions of the target mRNA were selected. Medium GC-content non-silencing siRNA (Invitrogen) was transfected as a negative control. Transfections were performed at the onset of the serum starvation period using Dharmafect I and Accell medium (Dharmacon, Horizon Discovery, Cambridge, UK). To transfect NRVM cultured in 12-well dishes, 200 pmol siRNA duplexes were made up to 100 µl total volume in Accell media and the solution mixed by pipetting. In a separate tube, 6 µl Dharmafect I was added to 94 µl Accell medium and mixed. After incubation for 5 min at room temperature, the tubes were combined, mixed and incubated for a further 20 min at room temperature. During this incubation, NRVM culture medium was replaced with 800 µl pre-warmed Accell medium. At the end of the 20 min incubation, transfection complexes were added to the cultures in a drop-wise manner and incubated with the cells for 6 h. Post-transfection, Accell medium was replaced with fresh maintenance medium and the remainder of the serum starvation period carried out.

1. Arthur, J.S.; Cohen, P. MSK1 is required for CREB phosphorylation in response to mitogens in mouse embryonic stem cells. *FEBS Lett* **2000**, *482*, 44-48, doi:10.1016/s0014-5793(00)02031-7.
2. Wiggin, G.R.; Soloaga, A.; Foster, J.M.; Murray-Tait, V.; Cohen, P.; Arthur, J.S. MSK1 and MSK2 are required for the mitogen- and stress-induced phosphorylation of CREB and ATF1 in fibroblasts. *Mol Cell Biol* **2002**, *22*, 2871-2881, doi:10.1128/MCB.22.8.2871-2881.2002.
3. Liu, W.; Zi, M.; Jin, J.; Prehar, S.; Oceandy, D.; Kimura, T.E.; Lei, M.; Neyses, L.; Weston, A.H.; Cartwright, E.J.; et al. Cardiac-specific deletion of mkk4 reveals its role in pathological hypertrophic remodeling but not in physiological cardiac growth. *Circ Res* **2009**, *104*, 905-914, doi:10.1161/CIRCRESAHA.108.188292.

4. Archer, C.R.; Robinson, E.L.; Drawnel, F.M.; Roderick, H.L. Endothelin-1 promotes hypertrophic remodelling of cardiac myocytes by activating sustained signalling and transcription downstream of endothelin type A receptors. *Cell Signal* **2017**, *36*, 240-254, doi:10.1016/j.cellsig.2017.04.010.
5. Thienpont, B.; Aronsen, J.M.; Robinson, E.L.; Okkenhaug, H.; Loche, E.; Ferrini, A.; Brien, P.; Alkass, K.; Tomasso, A.; Agrawal, A.; et al. The H3K9 dimethyltransferases EHMT1/2 protect against pathological cardiac hypertrophy. *J Clin Invest* **2017**, *127*, 335-348, doi:10.1172/JCI88353.
6. Higazi, D.R.; Fearnley, C.J.; Drawnel, F.M.; Talasila, A.; Corps, E.M.; Ritter, O.; McDonald, F.; Mikoshiba, K.; Bootman, M.D.; Roderick, H.L. Endothelin-1-stimulated InsP3-induced Ca2+ release is a nexus for hypertrophic signaling in cardiac myocytes. *Mol Cell* **2009**, *33*, 472-482, doi:10.1016/j.molcel.2009.02.005.
7. Drawnel, F.M.; Wachten, D.; Molkentin, J.D.; Maillet, M.; Aronsen, J.M.; Swift, F.; Sjaastad, I.; Liu, N.; Catalucci, D.; Mikoshiba, K.; et al. Mutual antagonism between IP(3)RII and miRNA-133a regulates calcium signals and cardiac hypertrophy. *J Cell Biol* **2012**, *199*, 783-798, doi:10.1083/jcb.201111095.
8. Dries, E.; Santiago, D.J.; Gilbert, G.; Lenaerts, I.; Vandenberk, B.; Nagaraju, C.K.; Johnson, D.M.; Holemans, P.; Roderick, H.L.; Macquaide, N.; et al. Hyperactive ryanodine receptors in human heart failure and ischaemic cardiomyopathy reside outside of couplons. *Cardiovasc Res* **2018**, *114*, 1512-1524, doi:10.1093/cvr/cvy088.
9. Vandesompele, J.; De Preter, K.; Pattyn, F.; Poppe, B.; Van Roy, N.; De Paepe, A.; Speleman, F. Accurate normalization of real-time quantitative RT-PCR data by geometric averaging of multiple internal control genes. *Genome Biol* **2002**, *3*, RESEARCH0034, doi:10.1186/gb-2002-3-7-research0034.
10. Livak, K.J.; Schmittgen, T.D. Analysis of relative gene expression data using real-time quantitative PCR and the 2(-Delta Delta C(T)) Method. *Methods* **2001**, *25*, 402-408, doi:10.1006/meth.2001.1262.
11. Alessi, D.R. The protein kinase C inhibitors Ro 318220 and GF 109203X are equally potent inhibitors of MAPKAP kinase-1beta (Rsk-2) and p70 S6 kinase. *FEBS Lett* **1997**, *402*, 121-123, doi:10.1016/s0014-5793(96)01510-4.
